# Arterial stiffness and vascular aging: mechanisms, prevention, and therapy

**DOI:** 10.1038/s41392-025-02346-0

**Published:** 2025-09-01

**Authors:** Maximilian Jonathan Herzog, Patrick Müller, Katharina Lechner, Marvin Stiebler, Philipp Arndt, Matthias Kunz, Dörte Ahrens, Alexander Schmeißer, Stefanie Schreiber, Ruediger C. Braun-Dullaeus

**Affiliations:** 1 Division of Cardiology and Angiology, University Hospital Magdeburg, Magdeburg, Germany; 2 Semmelweis University Budapest, Budapest, Hungary; 3 German Center for Neurodegenerative Diseases (DZNE) Magdeburg, Magdeburg, Germany; 4 Center for Intervention and Research on Adaptive and Maladaptive Brain Circuits Underlying Mental Health (C-I-R-C), Magdeburg, Germany; 5 German Center for Mental Health (DZPG), Magdeburg, Germany; 6 Institute of Epidemiology, Helmholtz Zentrum München, German Research Center for Environmental Health, Neuherberg, Germany; 7 DZHK (German Centre for Cardiovascular Research), partner site Munich Heart Alliance, Munich, Germany; 8 Division of Neurology, University Hospital Magdeburg, Magdeburg, Germany

**Keywords:** Cardiology, Cardiovascular diseases

## Abstract

Cardiovascular diseases are the leading cause of morbidity and mortality worldwide. The central underlying mechanisms of cardiovascular diseases are vascular aging and associated arterial stiffness. Arterial stiffness is characterized by structural (e.g., tunica media calcification, alterations in vascular smooth muscle cells, and fibrosis) and functional (e.g., loss of *Windkessel function*, elevated pulse pressure, and development of isolated systolic hypertension) vascular changes that cause microvascular dysfunction and end-organ damage (e.g., heart failure, vascular dementia, hypertensive retinopathy, and chronic kidney disease). Current research indicates that arterial stiffness is an independent risk factor for cardiovascular diseases and represents a potential target for personalized prevention and therapeutic approaches. In this review, we summarize the pathophysiological mechanisms of vascular aging and arterial stiffness, outline the resulting end-organ damage, present different methods for the measurement of arterial stiffness, highlight the potential role of prevention and therapy, and provide future perspectives for arterial stiffness research. The purpose of this review is to provide a state-of-the-art interdisciplinary and translational approach to arterial stiffness, highlighting unique pathophysiological mechanisms (e.g., perivascular adipose tissue, extracellular vesicles), clinical relevance, and future directions.

## Introduction

Cardiovascular diseases (CVDs) are the leading cause of death worldwide and contribute substantially to the global disease burden.^1^ In 2019, CVD caused 32% of all global deaths, corresponding to an estimated total of 17.9 million individuals.^2^ A wide range of disorders, including alterations of the cardiac muscle or the vascular system, are linked to CVD.^1^ The central underlying mechanisms of CVD are vascular aging and arteriosclerosis.^3^ Vascular aging occurs as a result of cumulative exposure to inherited and acquired risk factors and their interplay (epigenetics), leading to end-organ damage in various tissues/organs via endothelial and microvascular dysfunction, arterial stiffness, and atherosclerosis.^4^

**Fig. 1:**
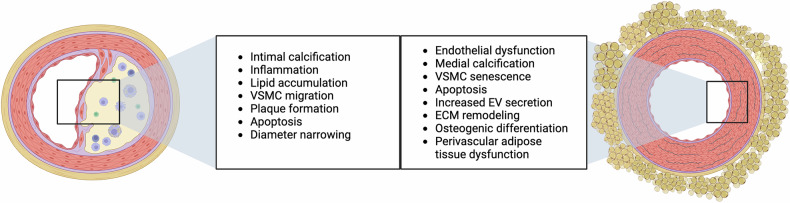
Schematic illustration of the structural and functional differences in atherosclerosis (left) and arterial stiffness (right). VSMC vascular smooth muscle cells, EV extracellular vesicles, ECM extracellular matrix. Created with BioRender.com

*Arteriosclerosis* is a collective term for different vascular diseases, including atherosclerosis, arterial stiffness, arteriolosclerosis, and Mönckeberg medial calcific sclerosis^5^ (Fig. 1).

*Atherosclerosis* is a degenerative disease of elastic and large muscular arteries manifesting as atheromatous changes in the tunica intima and thickening of the vessel wall via accumulation of plasma lipids (especially cholesterol) and connective extracellular matrix (ECM) (collagen fibers and proteoglycans) as well as the migration of cells (smooth muscle cells, monocytes/macrophages), which is accompanied by proliferation, calcification, ulceration, hemorrhage, and thrombosis formation.^5^ Various theories exist concerning the genesis of atherosclerosis at the cellular level. The most common theory is the response-to-injury hypothesis.^6^ Risk factors (hypertension, diabetes mellitus, smoking, and elevated lipids) cause both oxidative stress and minimal lesions at the high-pressure bifurcations of conducting vessels, which lead to an inflammatory response and the release of chemokines by endothelial cells. Subsequently, monocytes accumulate and invade the tunica intima and develop into macrophages. By phagocytosing chemically modified low-density lipoprotein (LDL), these cells become foam cells, which make up the largest part of the core of the plaque. Growth factors secreted by the local endothelium induce vascular smooth muscle cells (VSMCs) to migrate from the tunica media into the neointima, where they multiply. They additionally secrete the ECM, resulting in stabilization of the plaque. Certain processes, such as degradation of the fibrous cap by matrix metalloproteinases (MMPs), contribute to the instability of the plaque. Plaque rupture results in exposure of highly thrombogenic material to the bloodstream and subsequent thrombotic occlusion of the vascular lumen. The clinical correlate is an infarct (stroke, myocardial infarction).^7^

*Arteriolosclerosis* generally affects the arteriolar part of the arterial system and causes hyalinization within the tunica intima as well as hypertrophy of the tunica media.^8^ This process is particularly linked to hypertension and diabetes mellitus, among many other contributing factors.^5^

*Mönckeberg´s sclerosis* occurs in the tunica media and is disputed in the internal elastic lamina.^9^ This calcification process is frequently found in the muscular arteries of the extremities in elderly people with diabetes mellitus and chronic kidney failure,^10^ without remarkable narrowing of the arterial lumen.^5^

*Arterial stiffness* is characterized by structural as well as functional vessel wall changes that result from endothelial dysfunction and remodeling of the tunica media.^11–14^ This is followed by a loss of elasticity of large cushioning arteries, especially the aorta, and current research indicates that it is one of the earliest markers of vascular aging.^15^ In addition to the generation of reactive oxygen species (ROS), a series of events, including inflammation, calcification, elastin crosslinking, and the accumulation of collagens, occur, leading to increased arterial stiffness overall.^11^ Endothelial dysfunction, another key driver, is characterized by a proinflammatory state with reduced vasodilatory capacity and prothrombotic properties, which is caused mainly by the absence of nitric oxide and the increased generation of chemokines.^16^ These factors lead to greater stiffness and a downward spiral, leading to end-organ damage.

The aorta has the greatest elastin content within the arterial system and thus performs the important function of absorbing the pressure of systole and releasing it consistently in diastole so that pressure peaks can be dissipated and coronary perfusion can be increased.^17–19^ Arterial stiffness often progresses with age, causing fundamental alterations in central hemodynamics and significantly contributing to heart and kidney failure, cognitive decline, and other serious end-organ damage.

In particular, risk factors such as obesity (especially visceral adiposity), smoking, high blood pressure, diabetes mellitus, and dyslipidemia contribute significantly to this development.^20^ However, the causes and mechanisms of arterial stiffening are not fully understood. Currently, there are controversial chicken or egg discussions about arterial stiffness and cardiovascular risk factors.^21,22^ To some authors, arterial stiffness is a precursor to atherosclerosis, diastolic dysfunction, hypertension, and impaired cerebral/coronary blood flow, whereas others discuss arterial stiffness as a result of chronic risk factors such as hypertension and its own symptoms.^23^

Both atherosclerosis and arterial stiffness are systemic diseases and are linked to each other structurally and functionally. In a bidirectional relationship, arterial stiffness contributes to the progression of atherosclerosis with subsequent further stiffening of the arterial wall.^24–26^

In recent years, the assessment of arterial stiffness via pulse wave velocity (PWV) measurements has gained increased importance and recognition in clinical practice. Notably, the pulse wave was first graphically depicted and recorded by E.J. Marey with his specially revised sphygmograph in 1863 but lost importance owing to the technically less demanding measurement of blood pressure with an upper arm cuff, established by Riva Rocci in 1896. Since the 1970s, PWV has regained its reputation as a prognostic parameter.^27–29^

Today, the measurement of PWV has become a focus because it allows personalized risk assessment independently of blood pressure. Several studies have shown that arterial stiffness is a predictor of future cardiovascular events such as stroke or myocardial infarction.^30,31^ Moreover, arterial stiffness seems to predict CVD independent of traditional risk factors.^32^ Current research highlights the relevance of arterial elasticity/stiffness in vascular aging and cardiovascular diseases.^33^ However, the underpinning mechanisms are not fully understood. To date, there is no suitable animal model for arterial stiffness.

Current developments in the clinical measurement of arterial stiffness using multiomic and deep phenotyping approaches in combination with artificial intelligence analysis will provide novel insights into the underlying mechanisms, identify specific pheno- and endotypes of arterial stiffness and facilitate the development of personalized prevention and therapy approaches.

In this review, we summarize (i) the pathophysiological mechanisms of vascular aging and arterial stiffness, (ii) outline the resulting end-organ damage, (iii) present different methods for the measurement of arterial stiffness, (iv) highlight the potential role of prevention and therapy, and (v) provide future perspectives for arterial stiffness research.

The purpose of this review is to provide a state-of-the-art translational approach to arterial stiffness, highlighting unique pathophysiological mechanisms (e.g., perivascular adipose tissue (PVAT), extracellular vesicles), clinical relevance, and future directions.

## Anatomy and physiology of the arterial vessels

**Fig. 2:**
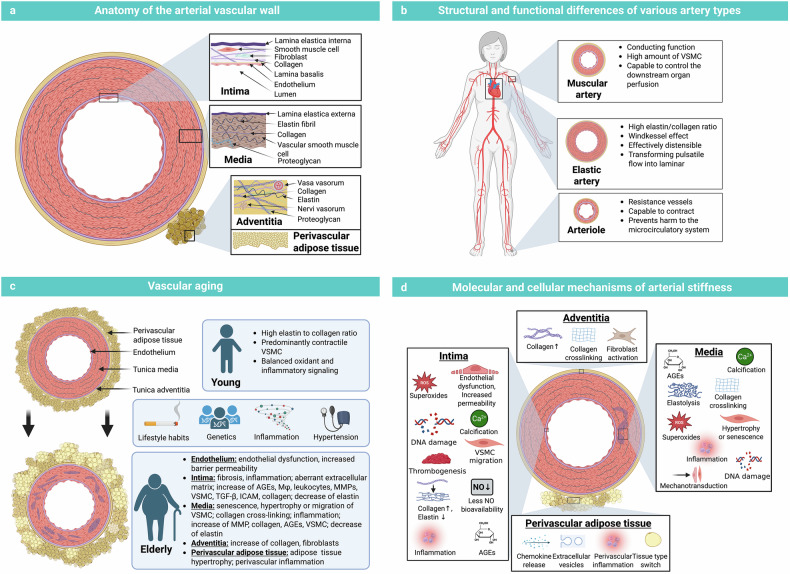
**a** Anatomical structure of the vascular wall from a cross-sectional view. The vascular wall is distinguished into the tunica intima, tunica media, and tunica adventitia/externa. Additionally, arteries are surrounded by PVAT. **b** Structural and functional differences in muscular arteries, elastic arteries, and arterioles. **c** Age affects arterial structure: we highlight the main alterations in the vascular wall leading to vascular aging and arterial stiffness. **d** Overview of the underlying molecular and cellular mechanisms of arterial stiffness in the different layers of the arterial system. Created with BioRender.com

Elasticity of the large arteries enables the vascular system to convert pulsatile blood flow from the heart to peripherally continuous flow. Thus, arterial elasticity (or vice versa stiffness) is characterized by structural and functional conditions of the vascular wall. Large arteries consist of three main layers: the tunica intima, tunica media and tunica adventitia, and surrounding perivascular adipose tissue (PVAT) (Fig. 2a). Several mechanisms in each layer affect the elasticity of the vasculature. However, the main determinant of arterial stiffness is the tunica media.

### Anatomical structure of the vascular wall

#### Tunica intima

The tunica intima consists of an endothelial monolayer and a basal lamina. Endothelial cells play a major role in the maintenance of vascular homeostasis, are crucial for vascular tone, and are involved in thrombolysis, coagulation, and inflammatory processes.^34^ A human adult has more than 10^12^ endothelial cells, which cover a surface area of nearly 1000 m^2^.^35^ The endothelium regulates the adhesion and infiltration of immune cells and controls vascular tone by releasing a balance of vasoconstrictive (e.g., endothelin-1) and vasodilatory (e.g., nitric oxide (NO)) factors. Additionally, endothelial cells synthesize several molecules, such as Von Willebrand factor (vWF), tissue factor (TF), prostacyclin (PGI2) and thrombomodulin (TM).^35^ Endothelial function is influenced by systemic (e.g., angiotensin II, extracellular vesicles) and local factors of the PVAT. PVAT contributes to endothelial function through the paracrine expression of adipokines, NO, and ROS.^36–39^ Classical cardiovascular risk factors, such as arterial hypertension, diabetes, smoking, and hypercholesterolemia, are associated with endothelial dysfunction.^40^

#### Tunica media

The tunica media consists predominantly of VSMCs, elastin fibers, collagen, and several cell‒matrix connections. In larger arteries, the cell layers of the tunica media are arranged to enable cushioning function. VSMCs are highly functionally relevant for blood pressure regulation and maintenance of vascular tone.^41^ Owing to its structural and functional components (e.g., elastin, collagen, and VSMC), the tunica media is crucial for vascular contraction and dilatation.^42^ Under physiological conditions, a contractile VSMC phenotype is characterized by low DNA synthesis activity and ECM production and is associated with contractile molecules such as alpha-smooth muscle actin, smooth muscle myosin heavy chain (SMMHC/SM-MYH), and smooth muscle protein 22-alpha (SM22α).^41,43^ High intracellular myofibril content and repeated contraction and relaxation of VSMCs contribute to vascular function.^44^

The composition of the tunica media changes with increasing distance from the heart, with reduced elastin and an increased proportion of smooth muscle cells. In this context, two main types of arteries can be defined: elastic central arteries (e.g., the aorta and pulmonary arteries) and muscular arteries (e.g., the femoral artery). Because of the reduced elastin content and increased VSMC proportion, peripheral arteries are stiffer, resulting in hemodynamic consequences (e.g., increased peripheral PWV).

#### Tunica adventitia

The tunica adventitia consists primarily of immune cells, fibroblasts, a collagen-rich ECM, and connective tissue with the vasa vasorum, lymphatic system, and nerve plexus.^45^ Collagen fibers are arranged longitudinally, and elastin fibers are ordered in a network.^46^ Functionally, the adventitia is able to damp and dissipate energy during pulse loading and prevent the vascular wall from overexpansion.^47^

#### Perivascular adipose tissue

Additionally, arteries are surrounded by PVAT, which is a heterogeneous tissue composed of adipocytes, fibroblasts, immune cells (e.g., macrophages), microvessels, and nerves. PVAT is responsible for up to 3% of total body adipose tissue.^48^ Historically, PVAT was thought to provide structural support to blood vessels. However, current research has demonstrated the fundamental structural and functional role of this tissue in vascular physiology. Via bidirectional crosstalk, PVAT is related to the vascular wall. It can release paracrine (direct effects on neighboring structures through diffusion) and/or endocrine adipokines, inflammatory factors (both pro- and anti-inflammatory), miRNAs, and other molecules influencing vascular homeostasis.^49^ Under physiological conditions, PVAT is associated with vasodilatory, anti-inflammatory, and antioxidant effects on the vasculature.^50^ Paracrine cells of the vasculature can influence the PVAT phenotype and modify secreted adipokines. Previous reviews highlighted the physiology of PVAT and summarized the main bioactive components in PVAT.^49,51,52^

### Windkessel function of large arteries

The large arteries are centrally responsible for conduits and cushion the function of blood flow. In systole, the stroke volume is ejected by the heart into the ascending aorta, whereas the inertia of the blood already occupying the lumen of the aorta creates a pressure wave that propagates to the periphery. The PWV refers to the speed of forward movement of the pressure waveform. Owing to the number of elastin fibers in the aorta, especially in the proximal part, the wall is capable of expanding, enabling the vessel to store the mechanical energy created by the blood and the pulse wave (cushioning function).^53^ After expansion, the blood is slowly released during diastole and enables sufficient cardiac perfusion. This process is commonly known as the *Windkessel function* and creates a more laminar and continuous flow (Fig. 5a).^54,55^ Elastin fibers in the arterial wall manage the distension under low-pressure circumstances, guaranteeing high compliance, whereas less extensible collagen fibers bear the tension under high pressure. Thus, a nonlinear pressure‒volume relationship is formed, in which blood pressure is a notable determinant for evaluating arterial stiffness (Fig. 5c).^53^ The pulse wave travels at a certain velocity down the aorta into the peripheral vessels and significantly exceeds the flow velocity of the blood. The wave itself is reflected at each bifurcation, increasing the upstream pressure pulse by superposition and acting as a mechanical stress at the bifurcation.^56^ The significant decrease in distensibility from the cushioning aorta to conducting vessels such as muscular arteries leads to an impedance change promoting wave reflection and pulse pressure elevation. This mechanism protects small arteries from harmful pulsatile stress due to wave reflection.^33^ With an intact aortal cushioning function, the PWV is slow, and the reflected wave returns in diastole, contributing to coronary perfusion. In a stiff aorta, a fast PWV returns in systole and promotes ventricular and vascular stress.^27^

The superposition of the pulse wave by the recurrent wave in the ascending aorta is called the augmentation pressure or augmentation index (Aix; proportion of augmentation pressure to pulse pressure; Fig. 5b). This parameter depends primarily on the vascular stiffness of large arteries and comprises endothelial function and distal microcirculation, total peripheral resistance (TPR), body size, systolic duration (heart rate), and sex.

The PWV depends on the intrinsic elastic properties of the arterial wall, especially the aorta, blood density, and vessel diameter.^53,57^ A simple equation for obtaining the PWV is utilized in daily practice.

$$c=\frac{{\rm{i}}}{\Delta t}$$

The pulse travel distance *i* and pulse travel time *t* can be approximated between 2 points, such as the carotid and femoral arteries.^54^

To quantify vascular age, including the condition of endothelial function and the *Windkessel* effect, the PWV can be approximated as follows:

$$c=\surd \frac{\kappa }{\rho }$$

Derived from the Moens–Koerteweg equation, where *κ* describes the volume modulus of elasticity, which decreases with greater extensibility and vice versa. The denominator contains the viscosity of the blood, which is expressed as *ρ*.

While Moens–Koerteweg describes the material properties of the arterial wall, Bramwell and Hill established their equation in 1929, monitoring wave speed as a function of arterial compliance and hence connecting the distensibility of the vessel with the propagation of pulse velocity.^27,58^

Otto Frank popularized the two-element *Windkessel* model in 1899, which describes TPR, (dynsec/cm^5^) and arterial compliance (C, [ml/mmHg]) as the main components. The first can be calculated using the mean arterial and venous pressure and cardiac output (CO) (*P*_ao,mean_ – *P*_ven,mean_/CO). The latter is the change in volume at a certain pressure, a measure of extensibility. Previous reviews have described methods for evaluating compliance and distensibility.^27^

The two-element model provides crucial information about the current cardiovascular condition. During diastole, a nonlinear pressure decay is displayed in an exponential curve, which can be approximated as the product of peripheral resistance and arterial compliance (Fig. 5b). However, the model was later improved by characteristic impedance and arterial inertance, as it did not match wave travel aspects properly.^59^

### Vascular tone

The endothelium, for instance, emerges as a central player in vascular tone regulation. It synthesizes and releases endothelium-derived relaxation factors such as prostaglandins, endothelium-dependent hyperpolarization factors (EDHFs), NO, and its counterpart endothelium-derived contracting factors.^60,61^ NO, which is released by endothelial cells, enables relaxation while activating soluble guanylyl cyclase (sGC) in VSMCs, which in turn initiates the production of cyclic guanosine monophosphate (cGMP).^60^ The main targets of cGMP are cGMP-dependent protein kinase Ια (GKΙα) and Ιβ (GKΙβ), resulting in numerous actions culminating in vascular relaxation and the inhibition of platelet aggregation.^62,63^ However, one underlying mechanism is the phosphorylation of Rho A, which inhibits Rho kinase, thereby activating myosin light chain phosphatase, which in turn leads to smooth muscle cell relaxation.^64^ Similarly, the phosphorylation of 1,4,5-inositoltrisphosphate receptor-associated cGMP kinase substrate, a protein located in the membrane of the endoplasmic reticulum, in a ternary complex initiates the inhibition of the IP_3_-induced release of Ca^2+^ from the endoplasmic reticulum, which promotes the relaxation of VSMCs.^62,63^

If endothelial function is disbalanced and the relaxation process is disturbed, arteries tend to gain more proinflammatory, prothrombotic, and vasoconstrictive properties.^61^

### Summary: Anatomy and physiology of the arterial vasculature

1. **Windkessel function and central hemodynamics** Large elastic arteries (particularly the aorta) attenuate the pulsatile output from the heart and release it continuously during diastole, thereby reducing the pulsatile load on peripheral vessels and preserving coronary perfusion. This cushioning (Windkessel function) mechanism helps reduce cardiac workload and supports coronary perfusion.
2. **Arterial layer structure and vascular compliance** The coordinated interplay of the tunica intima (endothelium), tunica media (comprising VSMCs, elastin, and collagen), tunica adventitia, and PVAT is fundamental for maintaining arterial elasticity and physiological hemodynamics.
3. **Endothelial homeostasis and nitric oxide (NO)** Endothelial cells modulate vascular tone through the synthesis of NO, which leads to smooth muscle relaxation via cGMP signaling. Even mild endothelial dysfunction (e.g., due to oxidative stress or inflammation) impairs this regulatory axis, facilitating arterial stiffening and predisposing individuals to hypertension.
4. **Perivascular adipose tissue as a regulatory interface** Under physiological conditions, PVAT exerts anti-inflammatory and vasodilatory effects through adipokines (e.g., adiponectin) on the arterial wall. However, metabolic disturbances (obesity, insulin resistance) and aging may convert PVAT into a source of inflammatory mediators, thereby accelerating arterial remodeling and contributing to the development of vascular stiffening.
5. **Pulse wave propagation and early indicators of vascular aging** The velocity and timing of the arterial pulse wave are closely linked to vessel distensibility. In stiff arteries, the pulse wave travels faster, and the reflected wave arrives in systole, elevating afterload and contributing to isolated systolic hypertension (ISH). An accelerated PWV reflects compromised arterial elasticity and is regarded as a critical early marker of vascular aging.

## Mechanisms of arterial stiffening

Vascular aging and arterial stiffness are based on multidimensional interactions of intrinsic (e.g., genetics, inflammation) and extrinsic (e.g., environmental) factors over the lifespan. They are associated with structural (e.g., VSMC proliferation, remodeling of the ECM, increased collagen deposition, collagen crosslinking, and elastin fragmentation) and functional (e.g., VSMC phenotype switching, proinflammatory signaling, endothelial dysfunction, and PVAT dysfunction) vascular wall changes (Fig. 2b, d). This development is a complex process driven by systemic factors (e.g., low-grade systemic inflammation, obesity) closely related to different vascular cells (e.g., endothelial cells, VSMCs), the ECM, and PVAT.^14,23^ From a spatial‒temporal perspective, endothelial dysfunction and ECM degradation are the first components of the pathophysiology of arterial stiffness.^41,65^

Central factors that increase arterial stiffness across the lifespan are endothelial dysfunction, a reduced elastin-to-collagen ratio and proliferation, and phenotypic switching of VSMCs.^65^ Biomechanical analysis of animal models has indicated that up to 50% of age-associated arterial stiffness is due to VSMCs.^66^ An overview of the underlying mechanisms can be found in previous reviews.^14,27,33,65,67–70^

Age-associated arterial stiffening is more pronounced in large arteries than in peripheral arteries. For example, aortic stiffness increases by approximately 70% from early to midlife (10–50 years of age), whereas peripheral artery stiffness increases by approximately 20%.^71^ The different age-related changes are based on structural and functional differences in central and peripheral arteries and must be taken into account when diverse methods for measuring arterial stiffness are used (e.g., measurements of aortal arterial stiffness vs. measurements of brachial–ankle stiffness; Fig. 2c).

### Chronological vs. biological vascular aging

Across the lifespan, several external and internal factors influence vascular aging processes. Age-related structural and functional vascular changes are caused by “cyclic stress” (repeated cardiac contractions cause hemodynamic pressure changes with the greatest impact on the heart near large arteries and pulsatile and circumferential stress). Over the lifespan, repeated pulsatile stress causes fragmentation of elastin, decreasing the elastin-to-collagen ratio, VSMC phenotype switching, and several other alterations resulting in physiologically increased arterial stiffness.^65,72^

Genetic and high-risk lifestyle factors (e.g., obesity, diabetes, smoking, and metabolic syndrome) can also accelerate the vascular aging process.^73^ In this context, differentiation between chronological and biological age is necessary.

Chronological age is defined as the time from birth to a certain point without considering life experiences, functional levels and/or disease-associated vulnerability. Consequently, people of the same chronological age can differ in their biological age.^74,75^ Biological age is affected by several internal (e.g., physical fitness level) and external (e.g., environment) factors and their interplay (epigenetics).^76,77^ Moreover, the aging process is heterogeneous across multiple organ systems (e.g., the brain, cardiovascular system, and musculoskeletal system).^78^ Biological age is clinically more important than chronological age is, and the incidence and prevalence of noncommunicable diseases (e.g., dementia, diabetes, cardiovascular diseases) and mortality are reduced.^77,79^ Clinically useful biomarkers for biological age are currently being developed.^80,81^

In the context of vascular aging, current research conceptualizes differentiating between early vascular aging (EVA), normal vascular aging (NOVA), and supernormal vascular aging (SUPERNOVA) phenotypes.^82^ Thus, the concept of EVA is highly relevant in the clinic. EVA is characterized by accelerated vascular aging and is associated with the early development of CVD.^83^ On the other hand, SUPERNOVAs can help to identify resistance and resilience mechanisms of aging and develop personalized prevention and therapy strategies.

Currently, in the randomized controlled prevent coronary artery disease trial (PRECAD), if LDL-cholesterol <70 mg/dL is maintained, strict control of glucose and blood pressure levels can prevent atherosclerosis in young, healthy adults (20–39-year-old participants without known cardiovascular disease).^84^ Additionally, preclinical data indicate potential effects of selected drugs (e.g., rapamycin and nicotinamide mononucleotide) in reversing vascular aging.^85,86^

### Molecular and cellular mechanisms of arterial stiffness

Historically, arterial stiffness has been associated primarily with alterations in the ECM, e.g., elastin fragmentation and a reduced elastin-to-collagen ratio. In contrast, current research highlights the fundamental role of endothelial cell function and VSMC plasticity and proposes several other important mechanisms.^33,51,65^ An overview of the molecular mechanisms of vascular aging is given below and summarized in Fig. 2d and Table 2.

#### Endothelial dysfunction

**Fig. 3:**
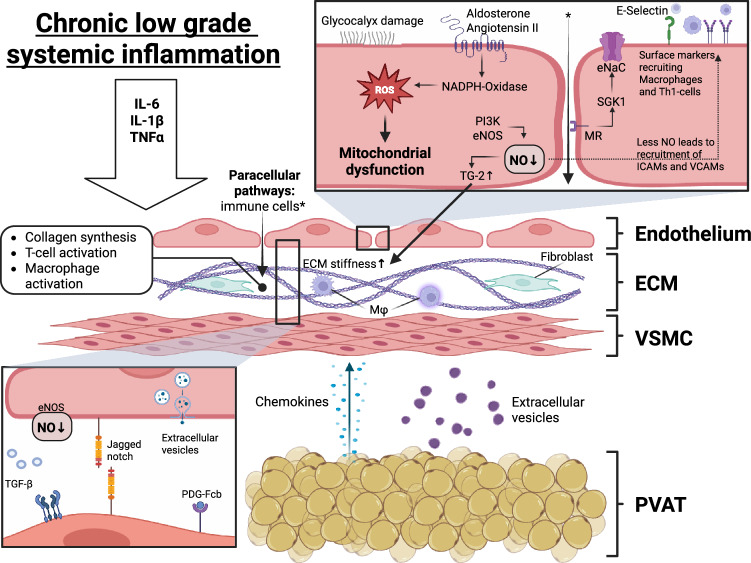
Low-grade systematic induction of endothelial dysfunction and resulting arterial stiffness. Chronic low-grade inflammation causes a plethora of endothelial molecular mechanisms, such as increased reactive oxygen species (ROS) and decreased activation of endothelial nitric oxide synthase (eNOS), resulting in reduced nitric oxide (NO) bioavailability, impaired barrier function, and transmigration of immune cells. The activation of the renin‒angiotensin‒aldosterone system (RAAS) increases through mineralocorticoid receptor (MR) NADPH oxidase, resulting in the generation of reactive oxygen species (ROS). Furthermore, mineralocorticoid receptor (MR)-induced activation of serum/glucocorticoid regulated kinase 1 (SGK1) causes increased Na+ influx through the endothelial Na^+^ channel (EnNaC). Elevated intracellular Na^+^ is associated with F-actin polymerization and endothelial stiffness. Decreased NO bioavailability causes the expression of adhesion molecules (e.g., VCAM and ICAM-1), resulting in the recruitment of monocytes and Th1 cells. Furthermore, decreased NO bioavailability causes transglutaminase 2 (TG2) activation, macrophage activation, and oxidative stress. TG2 is associated with ECM degradation and resulting ECM stiffness. Additionally, severe oxidative stress causes mitochondrial dysfunction and DNA alterations. Dysfunctional perivascular adipose tissue (PVAT) significantly contributes to the inflammatory state. While physiologically providing anti-inflammatory and vasodilatory signaling, age-associated PVAT releases several deteriorating molecules (e.g., leptin, chemerin, visfatin, resistin, and MCP-1). Created with BioRender.com

Animal and human studies have demonstrated that aging is a central cause of endothelial dysfunction.^87^ Cardiovascular risk factors (e.g., hypertension, diabetes mellitus, and truncal obesity) can accelerate this process and are associated with low-grade systemic inflammation, causing a specific sequence of endothelial dysfunction and tissue remodeling, e.g., ECM alteration and fibrosis^88^ (Fig. 2d; Fig. 3). Specifically, chronic low-grade systemic inflammation, characterized by increased levels of immune cells and cytokines (e.g., IL6, IL1β, and TNF-α), causes an elevated level of ROS and the upregulation of vascular cellular adhesion molecules (e.g., ICAM-1 and VCAM-1). ROS reduce NO bioavailability and impair NO/cGMP/protein kinase G (NO/cGMP/PKG) signaling, resulting in subsequent endothelial dysfunction.^89^ Barrier dysfunction and the recruitment of monocytes and Th1 cells following the upregulation of adhesion molecules cause vascular infiltration of leukocytes, resulting in ECM changes.

Low-grade systematic inflammation and the activation of the sympathetic nervous system are closely related.^90^ Activation of the sympathetic nervous system is observed in hypertension, diabetes mellitus, smoking, and obesity.^91–93^ Activation of the renin‒angiotensin‒aldosterone system (RAAS) increases nicotinamide adenine nucleotide phosphate hydrogen (NADPH) oxidase through specific endothelial mineralocorticoid receptors (MRs), resulting in elevated ROS. Furthermore, (MR)-induced activation of serum/glucocorticoid regulated kinase 1 (SGK1) causes increased Na^+^ influx through the endothelial Na channel (EnNaC). Elevated intracellular Na^+^ is associated with F-actin polymerization and endothelial stiffness.^94,95^ Moreover, current data indicate a potential fundamental role of endothelial dysfunction in obesity-associated arterial stiffness. In this context, studies have shown a bidirectional relationship between PVAT and endothelial cells.^51^ Under physiological conditions, adiponectin from adipocytes can increase the availability of NO and decrease the expression of adhesion molecules (e.g., ICAM-1 and VCAM-1).^96^ Age-associated dysfunctional PVAT synthesizes and secretes vasoconstrictive and inflammatory adipokines (e.g., leptin, chemerin, visfatin, resistin, and MCP-1), resulting in increased oxidative stress, reduced NO bioavailability, and endothelial dysfunction.^97^

The generation of NO is regulated by eNOS, but its expression can be restricted by the natural inhibitor asymmetric dimethylarginine (ADMA). Elevated plasma ADMA levels are found in patients with risk factors for atherosclerosis and extended intima media thickness (IMT).^11,98^

In addition, endothelial dysfunction is closely related to ECM and VSMC alterations, as endothelial cells modulate VSMC behavior via pleiotropic effects (e.g., eNOS derives NO and interactions via the ECM, extracellular vesicles, transforming growth factor beta (TGF-β), platelet-derived growth factor (PDGF), and Notch signaling).^99,100^

One underlying physiological impact of insulin is vasodilation via increased NO bioavailability to promote further glucose uptake by muscles, which is impaired under pathological conditions. If selective insulin resistance occurs, vascular dysfunction can be observed in both the macro- and microvasculature. This loss of function can likely be explained in part by insulin resistance in the phosphatidylinositol-3-kinase (PI3K) pathway, leading to increased signaling of the mitogen-activated protein (MAPK)-dependent subsystem and hence to an unbalanced vascular tone.^101^

In addition to NO, several other mediators, such as EDHF, are considered to take part in endothelium-attributed vasodilation responses. The key mechanism underlying the effect of EDHF on VSMCs is hyperpolarization; thus, relaxation occurs. Aging, hypertension, atherosclerosis, diabetes mellitus, hypercholesterolemia, and heart failure are linked to alterations in EDHF-mediated responses.^13^

Decreased telomerase activity in aged endothelial cells leads to defects on the luminal side of the vessel, allowing circulating platelets to dock, infiltrate, and initiate inflammatory processes. In addition, the repair capacity and migration/proliferation processes of endothelial cells are limited, culminating in increased endothelial cell (EC) contractility, permeability and intimal stiffening.^14,102,103^

In addition, elevated shear stress as a result of increased pulsatility in already stiff arteries leads to a reduction in the level of the messenger substance NO and a lack of cytoprotection against oxidative stress, which is potentially important for the progression of arterial stiffening. In contrast, high physiological pulsatility due to exercise in vessels with high compliance has been shown to increase endothelial nitric oxide synthase (eNOS) activity and improve endothelial cell survival and adhesion.^12^

Research by Leloup et al. demonstrated potential compensatory mechanisms of VSMCs (e.g., Ca^2+^ handling) to compensate for optimal central hemodynamics following endothelial dysfunction.^104^

In contrast, some mechanistic animal studies in C57Bl/6 mice have indicated that arterial stiffness is due to ECM and VSMC changes rather than endothelial dysfunction.^105^

#### Vascular smooth muscle cells

VSMCs play pivotal roles in the pathophysiology and progression of arterial stiffness, atherosclerosis, hypertension, aortic aneurysms, aortic dissection, and pulmonary arterial hypertension.^106^ During aging, VSMCs undergo changes in the organization of the cytoskeleton and their interaction with the ECM via integrins.^27^ VSMCs are considered one of the most diverse cell types in the vascular wall and are capable of phenotype switching. When initiated by several factors or pathways (e.g., inflammation, obesity, cytokines, miRNAs, and extracellular vesicles) and during aging, VSMCs can transition from a contractile phenotype to a more proliferative, migratory, and secretory phenotype (synthetic phenotype) or to a macrophage-like, nonprofessional phagocyte subset providing an inflammatory environment.^14,107–110^ A synthetic VSMC phenotype is associated with elevated osteocalcin (OCN), osteopontin (OPN), and bone morphogenetic protein 2 (BMP-2) levels and increased synthesis of ECM-influencing metabolites (e.g., MMPs).^41^ When triggered by milk fat globulin-E8 (MFG-E8), some VSMCs become more proliferative, while stress induces premature senescence (SIPS) through angiotensin II (Ang II) signaling, telomer shortening, or oxidative stress.^14,111,112^ Additionally, damaged endothelial cells release TNF-α, which triggers VSMC migration and increases MMP-9 levels, thus increasing elastin fragmentation. TNF-α is generally associated with VSMC phenotype switching induced by the RhoA/cell cycle pathway.^41^

Further signaling pathways associated with VSMC phenotype alterations include the TGF-β pathway, MAPK pathway, WNT pathway, and Notch pathway, which are excellently described in detail in previous reviews.^41^

PDGF and TGF-β are crucial growth factors for VSMC proliferation, ROS production, and fibrosis, resulting in ECM alterations (e.g., collagen synthesis). NO from endothelial cells can reduce VSMC proliferation and induce VSMC relaxation.^65^

Alterations in the TGF-β signaling pathway play crucial roles in different age-related morbidities, such as CVD, Alzheimer´s disease, and osteoarthritis.^113^ TGF-β1, the most functional pleiotropic isoform, enhances vascular fibrosis by enabling the expression of fibronectin, collagen type 1, and connective tissue growth factor (CTGF) via the activation of both the epidermal growth factor receptor (EGFR)/pp60c-src/MEK-ERK pathway and the Rho/ROCK-dependent SMAD2 pathway in VSMCs.^114,115^ Hence, vascular remodeling is initiated, leading to structural wall alterations and significant changes in mechanistic properties.^114^ Furthermore, TGF-β signaling is involved in multiple aspects of aging processes, such as cell proliferation, cell cycle regulation, ROS production, DNA damage repair, telomer shortening, the unfolded protein response (UPR), and autophagy.^113^

In primates, senescent VSMCs release a specific profile of proinflammatory mediators such as TGF-β, TNF-α, MFG-E8, matrix metalloprotease 2 (MMP2), and monocyte chemoattractant protein-1 (MCP-1) to surrounding cells, triggering a low-grade inflammatory process.^14,111,116^ This pattern of molecules is better known as the age-associated artery secretory phenotype (AAASP).^14^ An increased apoptotic rate of VSMCs in advanced age has also been reported, which is related to the pronounced activity of phosphodiesterase (PDE 5). This enzyme regulates the degradation of cGMP, influencing NO-dependent plasticity of the endothelium.^65^ Physiologically, NO from endothelial cells reduces VSMC proliferation and induces VSMC relaxation.^65^

Previous studies have described focal adhesion (FA) activation as a potential link between arterial stiffness and cell membrane mechanotransduction transmitted primarily by pulsatility.^65,69^ Cytoskeletal architecture and FAs are involved in cell‒matrix interactions. Furthermore, focal adhesion-associated molecules (e.g., vinculin and talin) are involved in the activation of the RhoA/Rho kinase pathway, resulting in vasoconstriction.^33^ RhoA is a small GTPase that modulates crucial cellular functions via its downstream target proteins ROCK1 and ROCK2. Both kinases are upregulated by interleukin 1β (IL-1β) and Ang II, thus inhibiting NO production in endothelial cells, enhancing ROS production and promoting CVD progression.^117,118^ The signals transmitted from the ECM to VSMCs significantly contribute to pathophysiology in normal and EVA cells through proliferation and alteration of plasticity. Additionally, current research indicates a direct connection between FA and the nuclear membrane. Thus, some FAs are directly linked with the VSMC nucleus through the actin cytoskeleton and could directly affect gene expression.^119^

In addition to vitamin K, cholecalciferol or vitamin D appear to play other important roles in the pathogenesis of arterial stiffening. Low vitamin D levels have been associated with increased PWV and AIx.^120^ Stress on endothelial cells and the absence of vitamin D lead to the free availability of vitamin D binding protein (DBP) and thus increased biochemical activity, followed by the proliferation and migration of VSMCs.^121^ Furthermore, the RAAS is influenced by vitamin D receptors; the absence of such receptors increases the production of renin and Ang II in mice. Consequently, cardiac hypertrophy, hypertension, and increased water intake have been observed.^122^ In addition, cholecalciferol is considered to play an immunomodulatory role by regulating the differentiation of lymphocytes and monocytes/macrophages. Thus, cytokines are released, further accelerating the recruitment of monocytes and the retention of cholesterol.^120^

Recently, Faleeva and colleagues focused on Sox9 (SRY-box transcription factor 9) as a strong regulator of VSMC chondrocytic differentiation and ECM-related gene expression. Occurring in calcified and aged vascular tissue, this transcription factor drives the senescent ECM phenotype, impacting stiffness, organization, and protein composition. ECM senescence leads to VSMC DNA damage and exit from the cell cycle. Sox9 initiates the increased deposition of LH-3 (procollagen-lysine, 2-oxoglutarate 5-dioxygenase 3) in the ECM via extracellular vesicles, further promoting ECM stiffness.^123^

#### Calcification

Calcification was detected in all 3 vascular wall layers. However, most relevant calcification occurs in the tunica media (especially around elastin fibers, elastocalcinosis) and within atherosclerotic plaques in the neointima.^124^ This process is mediated by chondrocyte-like cells from the bone marrow. In the adventitia, myofibroblasts and/or microvascular pericytes contribute to calcification. The tunica-mediated differentiation of VSMCs is significantly involved in this process. A disbalance between inhibitors of calcification (osteoprotegerin, osteopontin, etc.) and activators (fibroblast growth factor-23 (FGF-23)) and inflammatory cytokines has been suggested as the cause.^14,27^

Aged VSMCs also appear to behave like osteoblasts, producing more bone-like substrates, such as collagen 2. In addition, there is markedly elevated expression of calcification-promoting alkaline phosphatase in these cells. Especially in amyloidosis, calcification is accelerated by amyloid proteins and fibrils in the arterial wall.^14,27,67^

Matrix-Golgi protein (MGP) plays an extraordinarily important role in the mineralization process of vessels.^125^ This Gla protein is synthesized by chondrocytes, VSMCs, ECs, and fibroblasts in the arterial wall and undergoes two posttranslational modifications (carboxylation and serine phosphorylation) to function properly; the former is vitamin K dependent. Without MGP, mice die within 8 weeks of birth from ruptures of large vessels due to massive arterial calcification.^126^ Under normal circumstances, calcium-containing matrix vesicles are released by synthesizing VSMCs, while MGP protects against mineralization. However, at chronically high calcium levels, MGP is reduced, accelerating the calcification process.^127,128^ In animals, elastocalcinosis and elastin degradation by MMP-9 have been found upon treatment with vitamin K antagonists.^129^ The incorporation of circulating MGP as a biomarker is particularly interesting. Correlations between uncarboxylated MGP levels and the degree of calcification or cardiovascular disease have already been demonstrated.^125,130^

#### Renin Angiotensin Aldosterone System

The RAAS itself is strongly connected to the initiation and progression of atherosclerosis and arterial stiffness.^131–134^ Ang II induces the activation of nuclear factor kappa-light-chain-enhancer’ of activated B cells (NFκB) and consequently the expression of IL-6, MCP-1, and TNFα thus enhancing vascular inflammation.^131,135^ A positive feedback loop in injured arteries creates a vicious cycle via Ang II signaling. Vascular inflammation leads to the recruitment of inflammatory cells, which in turn generate more Ang II, further promoting the inflammatory process. Aldosterone also has proinflammatory and profibrotic properties, leading to vascular remodeling.^133^ Additionally, the RAAS triggers ROS generation by increasing the expression and activation of NADPH and mitochondrial protein kinase 1 as well as ATP-dependent potassium channel opening.^131,136^ In turn, ROS augment the expression of AT-1 (angiotensin-1) receptors, providing another positive feedback loop.^131^

#### Inflammation

The local proinflammatory milieu contributes immensely to the process of arterial aging, which is driven mainly by pathological alterations in VSMCs and ECs. In turn, inflammation further modulates the signaling of these cells, activating a downward spiral.^14,137^ Senescent cells belonging to these subtypes secrete a special pattern of inflammatory chemokines and molecules mentioned above as AAASP. Low-grade inflammation, which is strongly promoted by obesity^23^ and hypertension,^138^ accelerates arterial stiffness. One underlying mechanism is the direct reduction in eNOS caused by inflammatory mediators (e.g., TNF-α, IL-6, and CRP), which are usually elevated under inflammatory conditions.^139^ Consequently, the proinflammatory matrix and cellular modifications lead to the progression of hypertension and atherosclerosis.^14^

The continuous adjustment to aging alterations throughout a lifetime causes chronic physical and psychosocial stress. This so-called allostatic load induces the activation of the RAAS, the sympathetic nervous system, and the release of endothelin-1 (ET-1), triggering a proinflammatory process, which is in turn characterized by the excessive secretion and accumulation of chemokines such as TGF-β, MCP-1, MMPs and MFG-E8 and the activation or inactivation of various transcription factors such as SIRT-1 or NFκB .^14,67^

In particular, ROS drive inflammation via a variety of different modes and prominently emerge in aged arterial walls. The imbalance of NADPH oxidase and dismutase provides a pathologic and molecular background. Oxidase expression is triggered especially by high Ang II and ET-1 levels, which in turn aggravate endothelial dysfunction and vessel wall stiffening via proinflammatory markers.^14,67,103^

One important consequence caused by an array of inflammatory chemokines is DNA damage, which significantly accelerates vascular aging.^14,33,140^ Ataabadi and colleagues employed mice with a loss of function in the DNA repair endonuclease excision repair cross complement 1 (ERCC1) to display DNA damage and its nonatherosclerotic aging effects in the endothelium and VSMCs. Focusing on the latter, smooth muscle-selective genetic removal of ERCC1 deteriorated the subcutaneous microvascular dilatation capability and increased carotid stiffness due to impaired NO-cGMP signaling.^140^

Chronic viral infection is considered a contributor to pathological vascular aging, but its potential as a key driver cannot be ruled out. The herpetic cytokine virus (CMV), which presumably modifies VSMC function, causes inflammation, and leads to fibrosis of the tunica media, is suspected here.^141,142^

#### Perivascular adipose tissue signaling

Under physiological conditions, PVAT is associated with vasodilatory, anti-inflammatory, and antioxidant effects on the vasculature. Adipocytes can secrete several anticontractile adipokines, such as adiponectin, NO, and hydrogen sulfide (H2S), and anti-inflammatory cytokines, such as IL-10, prostacyclin, and TGF-β.^51^ However, aging is associated with alterations in the vasodilatory and anti-inflammatory effects of PVAT. Across the lifespan and accelerated under pathological conditions (e.g., obesity), there is increasing immune cell infiltration in PVAT, resulting in perivascular inflammation. T cells and macrophages subsequently release inflammatory cytokines (e.g., IL-17, TNF-α, and IL6), causing vascular dysfunction, arterial stiffness, and oxidative stress.^143,144^ Additionally, local inflammatory mediators from the vascular wall (e.g., IL6 and TNF-α) induce a change from storage to secretory cells in PVAT adipocytes.^145^ Age-associated dysfunctional PVAT synthesizes and secretes several vasoconstrictive and inflammatory adipokines (e.g., leptin, chemerin, visfatin, resistin, and MCP-1), resulting in increased oxidative stress, reduced NO bioavailability, and arterial stiffness. MCP-1 is associated with VSMC proliferation and the recruitment of macrophages.^146^ VSMC proliferation and macrophage migration are also caused by leptin, resistin and visfantin.^147^

Furthermore, PVAT-derived stromal cells can contribute to vascular remodeling via altered differentiation capacity and the loss of peroxisome proliferator-activated receptor gamma coactivator 1-alpha (PGC1α) in aged cells.^148^

#### Advanced glycation end products (AGEs)

Patients with impaired glucose tolerance or poorly treated or unrecognized diabetes mellitus have chronically elevated blood glucose levels. Under these circumstances, glucose binds to proteins. This process is called glycation and can lead to malfunctions of glycated proteins (e.g., stiffening of the arterial wall). In terms of the vasculature, there is an increased risk for glucose-mediated cross-links, especially when proteins with an expanded lifespan, such as carbohydrates and collagen, are involved.^149^

The aldehyde group of sugar reacts with an amino acid to form a Schiff base. Through further modifications, amadori-rearrangement products emerge, and oxidation, condensation and dehydration lead to AGE formation. These glycosylated proteins contribute to endothelial dysfunction and arterial stiffness and are involved in almost all steps of atherosclerosis.^7,149^ For example, macrophages are stimulated to release proinflammatory cytokines while vasoconstriction occurs, caused by an increased release of endothelin-1 and impaired production of NO. AGEs force endothelial cells to release elevated amounts of autocrine vascular endothelial growth factor (VEGF), leading to neovascularization and plaque instability. In addition, ROS are generated through various mechanisms. For example, high blood glucose levels result in extended glycolysis and thus increased oxidative phosphorylation. Super oxides and ROS are produced as part of a statistical process at any time.^7^

Interestingly, McNulty and colleagues have shown that AGEs are not only unique to diabetic patients. Hypertensive patients can also have elevated AGE blood levels in addition to increased PWV. A direct relationship between PWV and AGEs was considered, but AIx showed no significant relationship.^149^ Aging promotes AGE formation and collagen production via the activation of receptors for advanced glycation end products (RAGE), which further promotes the interaction of these molecules.^14^

#### Oxidative stress

The generation of superoxides and ROS represents a relevant mechanism in cellular senescence, vascular aging, arteriosclerosis, and arterial stiffening.

Superoxide anions (O2-) react with NO, forming peroxynitrate radicals (ONOO-), which are able to uncouple eNOS by scavenging tetrahydrobiopterin (THB), an essential cofactor for eNOS, hence accelerating superoxide production.^150,151^ In a hypertensive state, Ang II triggers the production of mitochondrial ROS (mtROS) and enables the mitochondrial permeability transition pore (mPTP) to leak mtROS into the cytosol.^152^ Subtypes of NADPH oxidase (NOX) are expressed in macrophages, VSMCs, endothelial cells, and fibroblasts, providing major sources of ROS. When O2 is reduced by NADPH, NOX expression is controlled by hormones, stress conditions, vasoactive agents, and cytokines.^150,153^ Oxidative stress leads to lipid oxidation, endothelial dysfunction, DNA oxidation, inflammation, and impaired plaque stability.^150^

As mentioned above, oxidative stress is involved in many pathophysiological pathways either by direct interference of these reactive molecules with essential cellular components or by initiating an inflammatory state leading to arterial stiffness and CVD progression.

#### Elastolysis

Elastin is thought to be formed during fetal development, and the human body is not capable of renewing or replacing it equivalently throughout life. Additionally, this fiber protein is subject to a constant process of degradation or transformation.^154^ In contrast, as an elastin counterpart, collagen is synthesized throughout life.^155^ Both molecules have different biophysical properties and occur in various proportions in all vascular walls, so the difference in renewability suggests a potential mechanism in vascular aging.^154^

The underlying mechanism primarily involves pulsatile continuous stress affecting the aorta and proximal vessels. A hypothetical heart beating at an average rate of 70 beats/min stretches the elastic lamellae of the vessels close to the heart via blood injection approximately 30 million times a year. Under this stress, material fatigue is expected, and stretchable elastin is replaced by stiff collagen. Natural rubber, which mimics the properties of a biological material, can become brittle after $8\times {10}^{8}$ cycles or after 30 years with this stress at the same heart rate and a 10% stretch. For peripheral arteries, which can only be stretched by approximately 3%, an equivalent of 100 years to reach the same condition is needed. Some fracturing and fraying of the elastin lamellae lead to significant stiffening of the aorta, which cannot be avoided and occurs in healthy individuals.^20,156,157^

Elastase and MMPs degrade interlamellar elastin fibers, minimizing the energy storage capacity and resilience of the aorta. Cleavage products, better known as elastin-derived peptides (EDPs), appear to be involved in the inflammatory and calcification processes of the arteries,^14,67^ promoting the progression of diverse vascular and metabolic diseases, such as atherosclerosis, nonalcoholic steatosis hepatis, thrombosis, arterial stiffness, and type 2 diabetes.^158–160^ One key pathway is mediated by the elastin receptor complex (ERC), which causes most of the biological effects of EDPs. NEU-1 (Neuramidase-1) is a major component of the ERC and has been identified as a potential target for inhibiting the detrimental effects of the ERC.^158^

The stability, stiffness, and extensibility of the arterial wall are predominantly determined by the ratio of elastin to collagen. Both molecules are normally kept in balance by a slow but stable remodeling process. MMPs, which are mainly responsible for this process, restructure the ECM by fragmenting elastin and disentangling collagen, thus altering its properties. These proteases can be overactivated by various processes, such as plasmin, thrombin, ROS, or MMP-MMP interactions.^11,161^ In response to a higher CO, e.g., obesity, elastin fibers are thinned, and thus, wall stress and strain increase so that additional collagen is mounted to bear the load.^162^

A particular role in the difference between men and women in vessel stiffness conditions is likely to be played by specific metalloproteases, involving the effect of cardiovascular protective estrogen. To prove this, in a study by Liu et al., mouse ovaries were removed while LDLR genes were switched off, and a high-fat diet was applied. Thus, endogenous estrogen production and a cholesterol-dependent effect could be excluded. Afterwards, exogenous estradiol was added, and the vascular status was assessed. Compared with those in the control group, both atherosclerosis and arterial stiffness were reduced by externally supplied estrogen. In addition, massive expression of MMP-12, a metalloprotease that has been associated with atherosclerosis, was discovered. Presumably, estrogen can block the synthesis of MMP-12 by macrophages. The induction of MMP-12 gene expression and pathophysiological mechanisms remain the subjects of current research.^155^

#### Coagulation

**Fig. 4:**
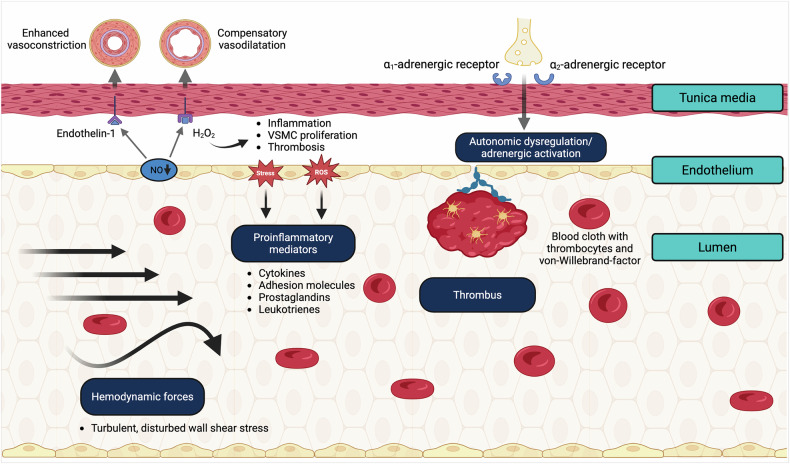
Interaction of endothelial dysfunction and hypercoagulability. Senescent endothelial cells and vascular smooth muscle cells are associated with increased synthesis of procovWFagulant (e.g., von Willebrand factor, fibrinogen) and anticoagulant (tissue factor pathway inhibitor) factors. In addition, hemodynamic forces, proinflammatory mediators and the autonomic state influence coagulation, resulting in increased endothelial cell permeability and perivascular inflammation. Created with BioRender.com

Endothelial dysfunction is associated with increased hypercoagulability (Fig. 4).^163^ The tunica intima plays a pivotal role in maintaining blood fluidity and barrier function. Arterial stiffness, local blood flow, and shear conditions influence blood cells, especially thrombocytes. Additionally, senescent endothelial cells and VSMCs are associated with increased synthesis of procoagulant (e.g., vWF, fibrinogen) and anticoagulant (tissue factor pathway inhibitor) factors. In particular, fibrinogen can induce endothelial cell permeability, increase vascular reactivity, and enhance perivascular inflammation.^164,165^

#### Cellular senescence and DNA alterations

Cellular senescence is a hallmark of vascular aging^166–169^ and is characterized by oxidative stress, cell cycle arrest, DNA alterations, proteostasis, telomere shortening, and mitochondrial dysfunction.

DNA integrity is constantly challenged by radiation, chemicals, or endogenous metabolic products. In particular, oxidative stress can induce DNA damage and suppress telomerase activity.^166^ Interestingly, endothelial cells seem to have less DNA repair capacity than other cell types do.^170^ Genomic instability, including chromosomal aneuploidies, somatic mutations, copy number variations, oxidative stress-induced DNA damage, and telomer shortening, significantly promotes vascular aging.^171^ Cell cycle regulators (e.g., retinoblastoma protein tumor suppressor activation and p53/p21) can enhance the inflammatory state and growth factors, resulting in structural and functional vascular wall changes.^172^

However, impaired protein homeostasis further aggravates the aging process. The downregulation of 70 kilodalton heat shock protein (HSP70) in vascular tissue has been linked to increased protein misfolding and aggregation activity.^72,173^ Another approach addresses protein disposal with respect to the dysregulation of autophagy.^72,174^

Mitochondrial ROS production caused by a dysfunctional electron transport chain or potential upregulation of the adapter protein ph66Shc is an additional source of vascular oxidative stress and senescence.^72,175^ For example, ROS-induced mitochondrial DNA variants are correlated with vascular compliance and age-related resistant hypertension,^175,176^ as well as impaired expression of mitochondrial-derived peptides, such as humanin, with protective effects on endothelial function.^175,177^

Moreover, cellular senescence generally promotes inflammatory mediators, matrix-degrading enzymes, and apoptosis and is able to reduce NO bioavailability, resulting in endothelial dysfunction, vascular remodeling, and perivascular inflammation^172^ (Fig. 4). Senescent vascular cells are characterized by reduced Sirt1, Klotho, and FGF21 levels. Previous reviews have highlighted the molecular mechanisms of cellular senescence and arterial stiffness.^167^

#### Genetic factors

Exome sequencing and genome-wide association studies have linked some gene variations to arterial stiffening. An example is the *CUL3* gene, which encodes Cullin3, a part of a ubiquitin ligase. Insufficiency of this enzyme causes reduced degradation of RhoA and increased RhoA/ROCK signaling, which promotes hypertension and arterial stiffening.^178^ Mutation of this gene has been shown to reduce the production of sGC. This results in endothelial dysfunction due to impaired production and activity of cGMP.^179^

Genetic variations in the *APOA5* gene have been associated with increased PWV, particularly in patients with decreased plasma HDL-C and adiponectin levels. These factors are independent parameters of arterial stiffness in type 2 diabetics and patients with elevated blood pressure.^180,181^

The mitochondrial genome also seems to play an important role, since the Framingham heart study revealed a correlation between an aberrant variant in the nicotinamide adenine dinucleotide phosphate (NADP) dehydrogenase subunit 5 gene and microvascular function.^182^

Additionally, evidence is growing that a connection between genetic variations in angiotensin receptor 1, left ventricular hypertrophy (LVH), and arterial stiffness exists. The likely pathomechanism is based on an alteration in Ang II receptor affinity.^183^

#### Epigenetics

Epigenetic modifications of histones can lead to impaired gene transcription. Reversible epigenetic factors appear to have a significant influence on arterial stiffening.^69^ A key mechanism appears to be hypomethylation of the promoter region of the *CIB2* gene on chromosome 15q25.1, which is associated with a single-nucleotide polymorphism (SNP). *CIB2* belongs to the *CIB1* family and has high structural similarity to calcineurin B and *CIB1*, which regulate intracellular calcium homeostasis.^184^ Extended expression of this gene resulted in significantly lower PWV.^69,185^

In a study of stroke-prone Dahl salt-sensitive rats, exposure to sodium intake and PWV was monitored after 3 and 6 weeks, while age and hypertension were excluded as influencing factors. Immunohistofluorescence analysis revealed significantly increased levels of a histone acetyltransferase (Ep300), a histone deacetylase (HDAC3), and a histone methyltransferase (Prmt5) in the left common carotid artery and aorta. Larger amounts of proteins were detected in all wall layers, indicating a clear progression of vascular aging. There was a detectable change in homeostasis in endothelial, smooth muscle, and adventitial cells. This study revealed some consequences of salt consumption on epigenetics, the ECM, and endothelial cell biology.^30^

It is well known that sirtuins play important roles in human aging and are established in common theories of aging. In the vasculature, they appear to exert beneficial anti-inflammatory and antioxidant effects as epigenetic modifiers.^181^ SIRT1 acetylates lysine 16 at histone 4 in the promoter region of plasminogen activator inhibitor-1 (PAI-1), leading to diminished transcription and generation of this molecule. In aged mice, this has been shown to improve endothelial relaxation and decrease PWV.^69,186^

Smoking also correlates with increased PWV in mice through interaction with sirtuins.^187^ Nicotine leads to increased expression of inducible NO synthase (iNOS), which generates peroxynitrite and hence damages the zinc-binding domain of SIRT1. As a result, more Yes-associated protein (YAP) is activated, and the ECM is remodeled. Increased amounts of collagen 1 and fibronectin are subsequently found in the aorta, measurably accelerating arterial stiffness.^187^

Regulatory miRNAs, which epigenetically affect gene expression, can also drive or delay vascular aging. They are able to interfere with gene transcription, which affects TGF-β and Ang II signaling or alters VSMC plasticity.^69,188^ These miRNAs influence many different pathogeneses, which are highlighted below.

#### Extracellular vesicles (EVs)

EVs are small, membrane-bound vesicles released by cells in a paracrine and/or endocrine fashion. In recent years, EVs have emerged as a subject of significant interest because of their ability to transport and deliver proteins, lipids, and microRNAs, facilitating intercellular communication.^189,190^ Across the lifespan, the synthesis and content of EVs change.^191^ Senescent VSMCs are associated with increased secretion of EVs.^192^ Research has demonstrated that fibronectin can increase VSMC EV secretion via activation of the β1 integrin/FAK/Src pathway.^193^

EVs are associated with vascular calcification, degradation of ECM components, and collagen crosslinking.^191^ However, the molecular mechanisms and role of EVs in vascular aging and arterial stiffness are not fully understood.

Animal studies highlight the potential function of EVs in vascular function, especially vascular tone, through NO synthase-dependent mechanisms.^194,195^

The PVAT-derived extracellular vesicle miR-221-3p mediates arterial stiffness via VSMC proliferation and migration.^196^ Furthermore, EVs are involved in the aggregation of misfolded proteins in the vascular wall and amyloidosis.^192^

### Modulating factors and comorbidities

Arterial stiffness is exacerbated by several cardiovascular risk factors, including obesity, hypertension, diabetes, smoking, metabolic syndrome, and chronic kidney disease. The partially different underlying mechanisms highlight the potential of specific phenotypes and endotypes.^124^ Identification of these endo- and phenotypes could help in the development of tailored preventive and therapeutic approaches for arterial stiffness.

#### Obesity

Arterial stiffness is more common in obese individuals and is an independent risk factor for cardiovascular events. Obesity-related arterial stiffness is more common in insulin-resistant people and women^23^ and is associated with cardiorenal metabolic syndrome. The central mechanisms of arterial stiffness in obesity are low-grade systemic inflammation, oxidative stress, activation of the RAAS and sympathetic nervous system, and hyperglycemia/hyperinsulinemia. Furthermore, obesity is associated with increased PVAT and a higher white-to-brown fat ratio,^197^ resulting in inflammatory and vasoconstrictive effects on the vascular wall. Obese adults exhibit increased levels of proinflammatory cytokines (e.g., IL-6, TNF-α, and IL-12) and an oxidative state in the PVAT.^97,198^ In particular, tissue macrophages produce proinflammatory mediators, resulting in endothelial dysfunction and VSMC proliferation.^51^

#### Hypertension

Arterial hypertension and arterial stiffness are pathophysiologically closely related.^27,199^ Previous studies have highlighted the chicken or egg dilemma.^22,200^ However, recent experimental and human data indicate that arterial stiffness can lead to arterial hypertension.^201^

The highlighted mechanisms of arterial stiffness in hypertension are mechanical stress, activation of the RAAS, endothelial dysfunction, and low-grade systemic inflammation.^202^

#### Air pollution

Current research highlights the role of air pollution as a potential cardiovascular risk factor.^203^ Recent data from Tasmin and colleagues suggest a relationship between long-term air pollution and arterial stiffness. A total of 2,387 subjects were examined for central blood pressure and stiffness parameters, such as brachial artery distensibility, compliance, and resistance, over a 3-year period. The results provide evidence that exposure to particles with aerodynamic diameters of <2.5 μm (PM2.5) and nitrogen dioxide (NO2) is associated with deteriorating stiffness parameters. The underlying mechanisms are not entirely understood; likely, compounds from air pollution lead to autonomous nervous system alterations, inflammation, a prothrombotic state, and oxidative stress.^204^

#### Diabetes

Hyperglycemia is associated with VSMC proliferation, AGE formation, ECM alterations (e.g., collagen crosslinking), and an increase in Ang II receptor expression. Insulin resistance can thereby enhance perivascular inflammation and collagen synthesis,^205^ as well as superoxide production and the impairment of NO synthase activation.^206^ Furthermore, it is worth considering whether advanced arterial stiffness contributes to diabetes mellitus through the transmission of harmful pulsatile stress into pancreatic vessels.^207,208^

In terms of prediction, arterial stiffness seems to be more useful for the determination of diabetes risk in patients than hypertension is.^209^

#### Chronic kidney diseases

One pivotal mechanism of arterial stiffness in CKD is calcification.^210^ Specifically, in dialysis patients, calcium phosphate levels are increased following arterial stiffening. In healthy adults, the precipitation of calcium is inhibited by several regulatory mechanisms (e.g., FGF-23 and Klotho).^124^ Furthermore, uremic toxins such as *p*-cresyl sulfate and indoxyl sulfate inhibit endothelial proliferation and increase oxidative stress, hence preventing NO production.^211^

However, it remains unclear whether CKD leads to arterial stiffness or vice versa, suggesting that renal dysfunction is both the cause and effect of arterial wall stiffening.^211,212^

#### Microbiota

Current research highlights the mechanistic link between cardiovascular diseases such as coronary heart disease and gut dysbiosis.^213–216^ Dysbiosis causes a low-grade inflammatory state and enables inflammatory pathways, hence providing the best conditions for atherosclerosis.^216,217^ Another approach involves gut microbiota-derived metabolites such as short-chain fatty acids (SCFAs), which are considered to have vasodilating properties.^218^ In contrast, another metabolite, trimethylamine N-oxide (TMAO), is capable of increasing ROS production as well as the secretion of IL-18 and IL-1β by binding to toll-like receptors (TLRs).^219^ Brunt et al. revealed a correlation between elevated TMAO and age in mice. Interestingly, the application of poorly absorbed antibiotics reversed endothelial dysfunction and arterial stiffness in this study.^220^

However, current human studies do not suggest a strong effect of the gut microbiota on arterial stiffness.^218,221^ A recent large cohort study by Cuadrat et al. revealed no association between arterial stiffness and the gut microbiota but suggested a potential role for individual gut microbiota components in the etiology of arterial stiffness.

#### Smoking

A recent meta-analysis revealed that acute smoking and vaping (including e-cigarette, vape pen or other electronic nicotine delivery systems (ENDSs)) exert a moderate negative effect on arterial stiffness. These effects are more likely related to functional than to structural changes.^222^ Nicotine, one of the main components responsible for addictive effects, causes the release of catecholamines, which promote the stiffening process through vasoconstriction and an increase in heart rate and blood pressure.

The consequences for chronic smokers remain controversial.^222–224^ Apparently, smoking cessation leads to a significant improvement in arterial stiffness.^222,225^ Mechanistically, smoking is associated with low-grade systemic inflammation and enables several harmful compounds to enter the human body. These factors can increase the production of ROS, which contributes to arterial stiffening. Jatoi and colleagues consider a potential cumulative effect of smoking and hypertension on arterial wall stiffening.^225^

#### Amyloidosis

Amyloidosis is characterized by the accumulation of misfolded amyloid protein and is a central pathophysiological feature of Alzheimer’s dementia and other diseases (e.g., hereditary transthyretin amyloidosis and cerebral amyloid angiopathy). The central risk factor for vascular amyloidosis (e.g., aortic medial amyloid, transthyretin amyloidosis, and amyloid light chain) is age.^226^ The role and mechanisms of amyloidosis are not well known.

Experimental data highlight the role of amyloid precursor protein (APP) and amyloid beta (Aβ) in vascular inflammation and vascular aging. Mechanistically, Aβ can induce ROS generation, resulting in endothelial dysfunction and perivascular inflammation.^227^

#### Chronic psychosocial stress (CPS)

Chronic psychosocial stress represents an increasingly recognized cardiovascular risk factor.^228^ Despite being implicated in several chronic diseases, particularly cardiovascular disorders, the mechanisms by which CPS impacts the cardiovascular system are not well understood. In general, psychosocial stress can be divided into acute psychosocial stress (APS) (e.g., terrorist attacks, sporting events) and chronic psychosocial stress (CPS) (e.g., loneliness, occupational stress, low socioeconomic status).^229^ Research has demonstrated that APS can increase arterial stiffness.^230,231^ The effects of CPS on arterial stiffness are not well known. However, the pathophysiological mechanisms (e.g., RAAS, low-grade inflammation) of CPS are partially involved in arterial stiffness and could indicate a potential link.

The CPS is associated with increased sympathetic nervous system activity, neuroendocrine activation of the RAAS, and several other molecular mechanisms.^228^ Via multiple pathways, it enhances proinflammatory chemokines and cytokines (e.g., TGF-β and MMPs) and activates or inactivates transcription factors (e.g., NFκB, Nrf2, and Sirt1).^14^

#### Hypoxic state

Vascular stiffness is a potential mechanism of increased morbidity and mortality in pulmonary diseases.^232^ For example, current research highlights increased arterial stiffness in patients with obstructive sleep apnea syndrome (OSAS). Intermittent hypoxia activates the autonomous nervous system as well as the RAAS and increases ROS production, leading to blood pressure elevation, atherosclerosis and arterial stiffening.^232^ OSAS patients also have increased levels of C-reactive protein (CRP), TNF-α, ICAM-1, IL-6 and IL-8, suggesting an inflammatory state. Several studies have provided evidence that continuous positive airway pressure (CPAP) treatment improves arterial stiffness in these patients.^233^

### Hemodynamic consequences of arterial stiffness

**Fig. 5:**
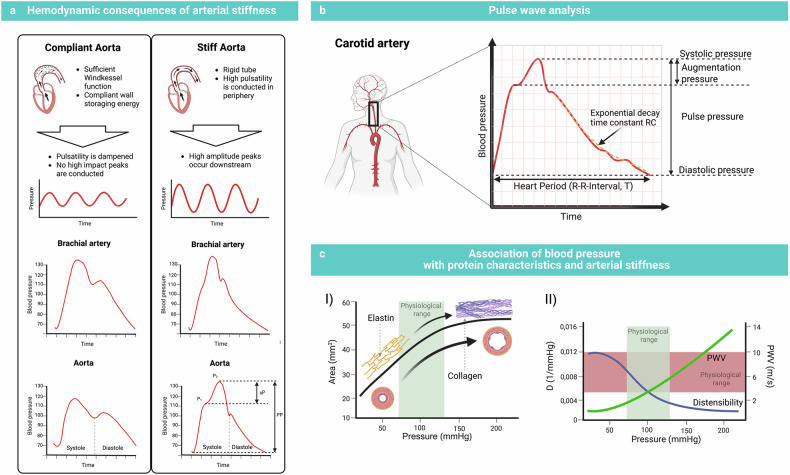
**a** Pulse wave analysis in a compliant versus a stiff arterial system. A compliant aorta is characterized by sufficient Windkessel function and dampened pulsatility. In contrast, a stiff aorta loses its Windkessel function, resulting in high pulsatility toward the periphery and a wave back unfavorably augmenting the central systolic pressure (augmentation pressure). **b** Pulse wave analysis of the carotid artery. The pressure decay during diastole can be approximated by the product of peripheral resistance (R) and arterial compliance **c**.^59^ **c** The first graph (I) depicts the proteins carrying the load at a certain pressure condition. Under hypertensive circumstances, collagen bears the load, so the vessel is not able to expand significantly, whereas under low pressure, elastin enables sufficient stretching qualities. The second graph (II) depicts distensibility properties in addition to the pulse wave velocity (PWV). Both curves function in a nonlinear and nonproportional manner, suggesting that hypertension leads to increased arterial stiffness^61^. Created with BioRender.com

Vascular aging and stiffening of the large arteries are associated with several adverse hemodynamic consequences (e.g., loss of Windkessel function, increased PWV, elevated pulse pressure, and microvascular alterations) (Fig. 5a). Organ-specific hemodynamic consequences are presented in each corresponding chapter.

Under physiological conditions in young adults, a compliant aorta has a fundamental cushioning function, the PWV is slow, and the reflected wave returns in diastole, hence contributing to adequate coronary perfusion. Pressure wave reflection emerges at several levels but is most pronounced at first-order bifurcations. Previous research has emphasized that a reflection wave is generated not only from a single reflection site but also from several individual reflections.^27^

Stiffening of the large arteries leads to an increase in the PWV, high forward wave amplitude and earlier return of the reflected wave. The earlier arrival of the reflected wave superimposes the incident pressure wave (Fig. 5a).

This condition leads to ISH and elevated pulse pressure, which is transmitted to the periphery and generates pulsatile stress as well as damage to the microvasculature.^234^ ISH is the main subtype of hypertension, with a prevalence of more than 75% in the elderly population aged 70 years or more. In the 5^th^ decade of life, the proportion of predominant diastolic primary hypertension caused by an extended TPR decreases, and ISH increases in importance.^235^ However, ISH may have different causes and does not always suggest increased PWV and advanced vascular aging. In slim, athletic adolescents, ISH can also be observed owing to the increased amplification of the pressure wave from the thoracic aorta to the measuring point in the brachial artery, but this phenomenon is an expression of sufficient elasticity of the arterial wall and does not require any therapy. Notably, this is a spurious case of hypertension. In this case, it can even be said that the higher the amplitude is, the better the physiologic condition.^235^

Furthermore, increasing evidence highlights a mutual relationship between macrovascular and microvascular dysfunction. Large artery stiffening leads to the transmission of deleterious pulsatility to small arteries, which in turn initiates a remodeling process, thereby increasing the TPR. This further enhances the pressure-driven stiffening process via a positive feedback mechanism. An elevated TPR leads to increased blood pressure and thus promotes arterial stiffness.^208,236^

### Summary: Mechanisms of arterial stiffening

1. **Endothelial dysfunction and low-grade systemic inflammation** *Systemic low-grade inflammation (e.g., IL-6, TNF-α, and MCP-1) impairs endothelial NO synthesis, promotes leukocyte infiltration, and increases oxidative stress. In turn, reduced endothelial NO bioavailability, excessive oxidative stress, and the release of proinflammatory cytokines promote arterial stiffening*.
2. **Vascular smooth muscle cell phenotype switching** *Chronic mechanical and inflammatory stimuli induce VSMCs to transition from a contractile to a proliferative phenotype. This phenotypic shift involves excessive ECM deposition, arterial calcification, and structural changes characteristic of advanced vascular aging*.
3. **Extracellular matrix remodeling and loss of elastic properties** *Dysregulated ECM turnover—marked by elastin fragmentation, collagen deposition, elastin fragmentation, and crosslinking (e.g., via advanced glycation end products)—undermines arterial compliance, leading to increased arterial stiffness and resulting in elevated pulse pressure. This ECM imbalance is a hallmark of vascular aging and a central cause of increased stiffness*.
4. **Epigenetic alterations and cellular senescence** *Age-associated epigenetic modifications (e.g., microRNAs, histone acetylation/methylation) as well as senescent endothelial cells and VSMCs (with reduced telomerase activity) secrete inflammatory mediators, accelerate elastin degradation, and promote calcification, further contributing to aberrant ECM remodeling, calcification, and chronic low-grade inflammation and subsequent accelerated arterial stiffening*.
5. **Modulating factors** Key comorbidities such as hypertension, diabetes, obesity, and chronic kidney disease exacerbate these mechanisms through inflammation, insulin resistance, and oxidative stress.

## Arterial stiffness and resulting end-organ damage

Physiologically, compliant large arteries are coupled to much stiffer peripheral arteries, providing a pronounced impedance mismatch and sufficient wave reflection. Additionally, the powerful cushioning function of the aorta enables a steady flow, keeping harmful pulsatility low. The second barrier maintaining pulsatile protection consists of vascular autoregulation and resistance vessel constriction. Thus, the pulsatile energy transmitted into the microcirculation can be limited.

**Fig. 6:**
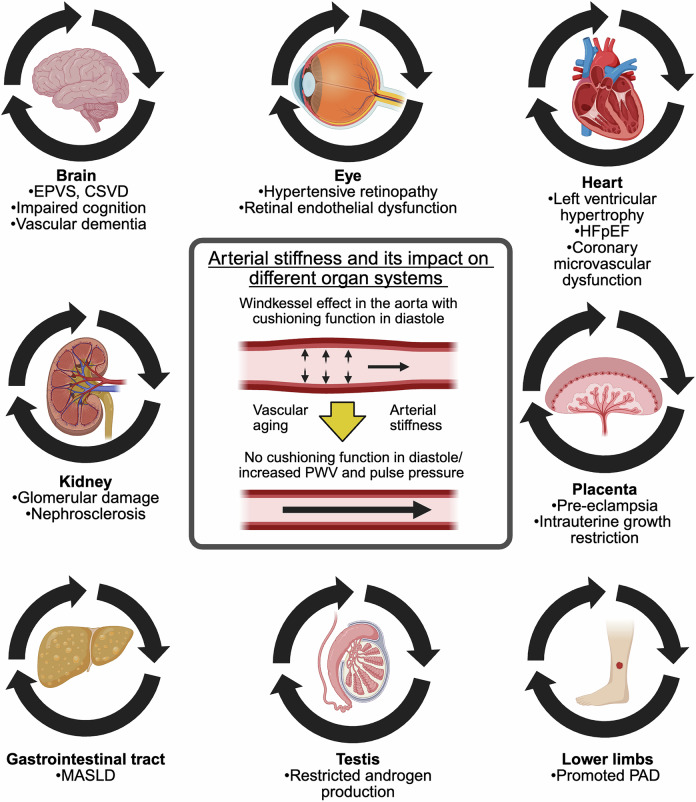
Effects of arterial stiffness on peripheral end organs and clinical manifestations. Arterial stiffening tends to create a vicious cycle in each organ. EPVS enlarged perivascular space, CSVD cerebral small vessel disease, HFpEF heart failure with preserved ejection fraction, MASLD metabolic dysfunction-associated steatotic liver disease, PWV, pulse wave velocity, PAD peripheral artery disease. Created with BioRender.com

Pathologically, arterial stiffness leads to ISH harming end organs via increased pulsatility. In particular, organs with high blood flow and low resistance properties are affected. Vascular remodeling and subsequent imbalance in perfusion occur further downstream (Fig. 6). Additionally, specialized tissues such as the heart and brain carry different pathological pathways, leading to different types of end-organ damage and vicious cycles that accelerate disease progression and deleterious effects.

### Heart

Vascular health is key to healthy aging. In modern times, reaching old age is increasingly limited by lifestyle habits. This connection is strongly related to cardiovascular diseases. There are two basic assumptions for pathological processes that can lead to final heart failure. On the one hand, the “cardiovascular continuum” was established in 2006 by Dzau and colleagues. Their publication addresses several risk factors, such as hypertension, diabetes mellitus, dyslipidemia, smoking, and truncal obesity, and their impact on the intimal atherosclerotic process with respect to genetic, molecular and cellular processes and potential therapies.^237^ Unfortunately, this approach analyzes primarily atherosclerosis and does not reveal the full range of pathological processes that contribute to cardiovascular failure and failure of other organ systems. The subsequent “vascular aging continuum” presented by O’Rourke et al. highlights the processes of vascular aging with respect to arteriosclerosis, especially in populations where atherosclerosis is not the predominant problem.^20,67^ For example, it affects special groups, such as elderly people in China^238^ and Japan, where the prevalence of atherosclerosis is low, or postmenopausal women in Western societies,^20^ with the latter likely due to hormonal impacts on the vasculature.^155^ Additionally, vascular risk factors are more strongly related to arterial stiffness in women than in men.^238^ Myocardial ischemia and myocardial failure in Western society are often caused by a mixture of atherosclerosis and advanced vascular aging; thus, vessel morbidity is considered a central factor.^20^

If aortic stiffness progresses, the heart is forced to exert much greater effort to propel blood in a rigid tube instead of a distensible aorta. The consequence is an elevation in pressure and PWV in the aorta during systole. A study with 111 subjects by Redheuil et al. reported an increase in the PWV of 7.6 m/s at the age of 20--70+ years, whereas O’Rourke and colleagues reported a pressure increase of approximately 20 mmHg at the age of 20--80 years.^20,157,239^ At the same time, however, the diastolic pressure decreases, as the slow blood release and pressure division of the dilatable aorta fail, so ISH occurs. The rapid pulse wave now returns in systole at the moment of maximum ejection, leading to an elevation in the systolic pressure of a further 30 mmHg and, in total, an increase in the aortic pulse pressure to 60–70 mmHg (3 times the normal value of a 20-year-old child) at 80 years of age.^20,157^

The consequence is an increase in afterload with subsequent left ventricular hypertrophy, which increases myocardial oxygen consumption. The enlarged heart contracts and relaxes more slowly, extending the systolic duration so that the reflected pulse wave is closer in time to the climax of ejection pressure, thus generating a vicious cycle. However, the shortened diastole and lowered diastolic blood pressure reduce the blood flow and oxygen supply to the heart. Atherosclerotic processes seem to occur independently of the pathomechanism described above.^157,240^ However, a connection is also discussed here, as atherosclerosis can be initiated and/or accelerated by the effect of increased shear stress on the endothelium.^241,242^

The extended afterload leads to persistent elevated filling pressures in the left ventricle and atrium; thus, remodeling of the myocardium is initiated, and later dysfunction occurs. This process can cause atrial fibrillation and heart failure with preserved ejection fraction (HFpEF). Because of remodeling, more collagen is stored; hence, the stiff myocardium is not capable of fully relaxing, so the end-diastolic volume and the stroke volume decrease.^243^

Another mechanism potentially connecting aortic stiffening and HFpEF is the impaired coupling function between the left ventricle (LV) and the aorta. If stiffness occurs in the proximal part of the aorta, the LV is not capable of complete longitudinal shortening in systole, resulting in adverse aortic longitudinal stretch and, in return, reduced aortic recoil. Thus, early diastolic filling is diminished, and the importance of late diastolic filling, which is caused mainly by atrial systolic constriction, is emphasized. Clinical evidence is based on a lower early diastolic mitral annulus tissue Doppler velocity.^162^

Taking microangiopathy into account, another publication reported that aortic stiffness in patients with nonobstructive coronary artery disease (CAD) elevates cardiac microvascular resistance and hence promotes coronary microvascular dysfunction.^244^

### Brain

Arterial stiffness, represented by elevated PWV, leads to cognitive impairment through several mechanisms.^27,67,245^ In particular, subcortical cognitive abilities such as executive and working memory functions are inversely associated with PWV.^246^ Previous reviews have highlighted the impact of vascular aging and arterial stiffness on cerebrovascular function and cognition.^247–250^

In the studies by Hajjar et al.^246^ and Bown et al.,^251^ PWV was shown to be a more predictive biomarker of later cognitive decline than was blood pressure. In their review, Albert de Roos and colleagues discussed the relevance of PWV measurement in relatively young and healthy adults in predicting dementia in all domains better than blood pressure evaluation does, emphasizing the importance of reducing arterial stiffness as a potential preventive/therapeutic target.^162^ Furthermore, there are proven associations between vascular aging and dementia, stroke, regional brain atrophy, and cerebral small-vessel disease, e.g., neuroimaging markers of microvascular disease, including white matter hyperintensities (WMHs), lacunar infarcts, cerebral microhemorrhages, and enlarged perivascular spaces (EPVSs).^27,252,253^ In particular, individuals aged 65 years or older display a significant correlation between central arterial stiffness, BP and cerebral blood flow and the resulting amount of white matter damage.^253^ There are also links between increased baseline aortic PWV and decreases in the volume of the hippocampal and occipital lobes.^251^

In the physiological state, blood is transported from the heart to the brain, and total flow is regulated by macrovascular vasodilation and vasoconstriction. This guarantees an adequate supply of nutrients and oxygen to the entire brain parenchyma and dampens pulsatile energy.^245^

The so-called Bayliss effect regulates blood flow through intramural smooth muscle cells independently of the body’s activation state and ensures constant blood flow, for example, to the kidney and brain. In contrast, the lungs behave in a pressure-passive manner; hence, the lumen of the vessels changes proportionally with intravascular pressure. However, there is only a limited pressure range for autoregulated organs. For the kidney, 70–160 mmHg was used, and for the brain, 50–120 mmHg was used. In addition to these limits, pressure-passive behavior also occurs.^55^ In organ systems with high flow and pronounced autoregulation, the microvasculature especially suffers when high pulse pressure waves can no longer be adequately attenuated in the context of arterial stiffening.^234,245^ Myogenic tone is crucial for the short-term regulation of blood flow and is generated by increased contractility following calcium influx into VSMCs.^253^ The starting point is the elevated tension of integrins in the ECM.^234,254^ Microvascular control mechanisms in the brain must, on the one hand, fulfill metabolic requirements while on the other hand, limiting blood flow to prevent damage from increased pressure, such as hyperfiltration, protein leakage, or edema.^234^ Local metabolic demands in response to fluctuations in neuronal activity are regulated via neurovascular coupling so that adequate regional blood flow is guaranteed. In contrast to macrovascular autoregulation of brain perfusion, this requires locally adaptable microvascular vasodilatation and vasoconstriction of parenchymal arterioles and even capillaries. These mechanisms are impaired in aging and hypertension.^255–257^

If advanced arterial stiffening occurs, the increased pulse pressure is transmitted to the microcirculation of organs and causes severe damage.^27,67,234,235^ One mechanism causing extended transmission is an impedance mismatch between the proximal aorta under stiff conditions and the carotid arteries, leading to decreased wave reflection between these arterial parts.^33,162^ Additionally, the pulse pressure increases as a consequence of ISH.^258^ This continuous stress of blood pressure peaks leads to loss of integrity of the BBB and, simultaneously, to remodeling of the vascular ECM in capillaries and arteries, increasing resistance and decreasing cerebral blood flow by extending the wall-to-lumen ratio.^234,253,259,260^ The cerebral microvascular remodeling process itself is accompanied by mechanosensitive signaling as the result of pathological hemodynamic stimuli as well as dynamic interactions between growth factors, cytokines, and vasoactive molecules generated by cells in the vascular wall.^253^ Furthermore, ongoing exposure to high pulsatility decreases cerebrovascular reactivity (CVR).^234,245^ This effect is significantly more progressive in carriers of the APOEε4 gene, which is linked to Alzheimer’s disease (AD).^245^ Arterial stiffness also leads to blood pressure lability, which, in terms of reduced CVR, causes frequent transient brain ischemia and consequently brain damage over time.^162^

There is evidence that alterations in the PP influence local blood flow regulation in terms of microvascular endothelial function, remodeling, and myogenic tone more sensitively in the brain than does the mean arterial pressure (MAP). If this hypothesis is correct, elevated vascular resistance caused by increased PP with constant or reduced MAP leads to accelerated perfusion lowering.^234^ Both the endothelium^261^ and the vasculature^262^ are susceptible to increased pulsatility, leading to hypertrophy of the arterial wall.^263^ For example, Ryan and colleagues demonstrated a connection between high pulse pressure and a decrease in acetylcholine (Ach)-dependent endothelium relaxation mediated by ROS and advanced shear stress, respectively.^264^ Additionally, the absence of NO most likely plays a crucial role under these conditions because its bioavailability is indirectly linked to high PP.^265^ One resulting mechanism is the inability to regulate cerebral blood flow adequately in response to the different demands of oxygen and nutrients that occur with neuronal activation.^72,266^

These ongoing remodeling measures only represent adaptations to changing circumstances (increased MAP and/or PP) and are intended to maintain reactivity with protection against barotrauma but cannot always guarantee the correct supply/demand ratio at rest, especially during rapid changes.^234^ Consequently, arterial stiffness is directly related to mild cognitive decline, vascular dementia, and Alzheimer’s disease.^267^

A meta-analysis by Pantoni and Garcia^268^ investigated the etiology of WMH: presumably, ischemia-related structural changes in the small intraparenchymal arteries and arterioles due to stroke risk factors, aging, and altered autoregulation cause these lesions. In addition, diabetes mellitus and hypertension also lead to remodeling processes of the small vessels and thus to narrowing or occlusion, resulting in small infarctions within the white matter. Alternative factors, such as disturbances in cerebral spinal fluid (CSF) circulation or failure of the barrier function of the BBB with activated, swollen astrocytes, probably contribute to the MRI abnormalities of WMH due to increased white matter water content and a decrease in myelin sheaths.^268^

Disturbances in BBB integrity and endothelial dysfunction are related to pericyte loss, which impairs the architecture of the cerebral microcirculatory network. Moreover, BBB disruption allows plasma compounds such as IgG, thrombin, and pathogen-associated molecular patterns (PAMPs) to enter the brain parenchyma, enabling microglial activation, neuroinflammation, synapse loss, and synapse dysfunction. Another consequence of high-pressure transmission into the cerebral microcirculation is a reduction in capillary density in the brain parenchyma, which is accompanied by further restriction of nutrient and oxygen supplies. Pericyte damage once again plays a role here, promoting a surplus of anti-angionic factors.^253^

A new concept is attributed to dysfunction of the glymphatic system, which was first discovered in 2013. This system, which is primarily responsible for waste disposal, removes soluble proteins and metabolites from the brain parenchyma and is considered a distributor for glucose, amino acids, lipids, and neurotransmitters.^269^ Arterial pulsation seems to be utilized as a driver to propel CSF from the subarachnoid space along penetrating arteries in perivascular spaces (Virchow–Robin spaces), leading to an exchange of CSF and ISF.^269,270^ The system is particularly effective during sleep and is repressed by 90% during wakefulness.^269,271^ The expression and incorporation of aquaporin-4 (AQP4) channels into the end feet of astroglial cells is required to allow the flow of CSF into the brain parenchyma (80,83). In wild-type mice, sleep disturbance provoked reduced activity of the glymphatic system, with impaired AQP4 expression.^272^

**Fig. 7:**
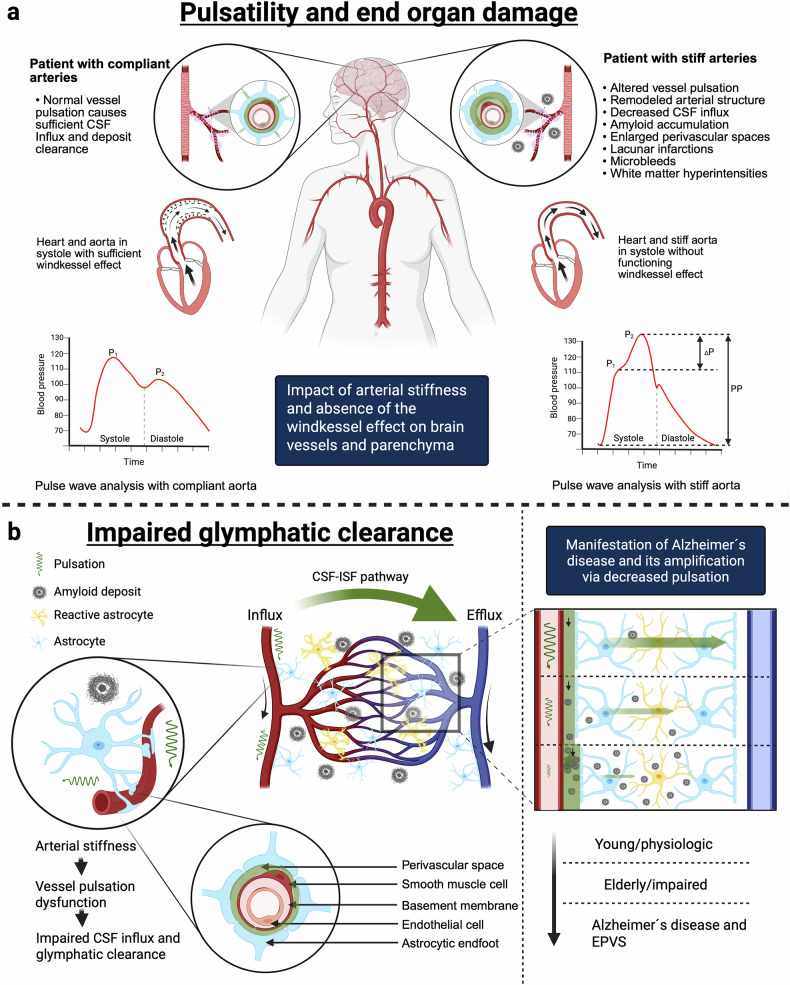
**a** Microscopic and macroscopic consequences of arterial stiffness and altered vessel pulsation on the brain parenchyma. The increased pressure peaks cause a remodeling process in the arterial wall structure; thus, pulsation is not transmitted into the peripheral microvasculature. This leads to a decrease in CSF influx, the deposition of amyloid proteins and the emergence of enlarged perivascular spaces. In addition, the remodeling process includes decreases in cerebral perfusion and small vessel disease, which can be visualized as microbleeds, white matter hyperintensities, and lacunar infarctions.^162,271^ **b** Impaired glymphatic clearance is presented on a microscopic scale. The altered pulsation caused by vessel remodeling and arterial stiffness leads to decreased CSF influx into the brain parenchyma and subsequent waste deposition, such as that of amyloid proteins. Stored amyloids cause inflammation and reactive astrogliosis, which promote neurodegenerative diseases. Additionally, we depicted the ongoing process in young individuals versus Alzheimer’s disease patients with enlarged perivascular spaces.^276^ CSF cerebrospinal fluid, ISF interstitial fluid, EPVS enlarged perivascular space, pp pulse pressure. Created with BioRender.com

As mentioned above, arterial stiffening leads to the transmission of harmful pulsation and subsequent remodeling of the arterial wall in small arteries, thus altering wall properties such as compliance and distensibility. Vessels are no longer capable of transmitting harmless pulse waves along perivascular spaces.^162,271^ Consequently, the driving force of the glymphatic system is weakened, leading to impaired perivascular CSF flow dynamics (Fig. 7). This has been demonstrated in models of chronic hypertension via MRI as well as via intravital imaging techniques.^273–275^ Increased amounts of misfolded or hyperphosphorylated proteins, as well as amyloidβ (Aβ), tau proteins, or α-synuclein, now accumulate in brain tissue and disrupt glymphatic function, promoting dementia and neurodegenerative diseases.^269,271,276^ Again, a vicious cycle can be surmised as the accumulation and aggregation of the aforementioned proteins, especially Aβ, leads to a blockage of draining the perivascular spaces (PVS) and to their enlargement, thus further reducing glymphatic influx.^269^ A direct link between carotid femoral PWV (cfPWV) and basal ganglia enlarged perivascular spaces (EPVS), as well as cerebral small vessel disease (CSVD), has been demonstrated in hypertensive individuals, linking arterial stiffness to cognitive deficits.^277,278^ EPVSs, particularly those occurring in the putamen and pallidum, are considered to serve as markers for vascular damage caused by high-pressure variability and flow pulsatility.^162,279^

Additionally, the distribution of misfolded and aggregating proteins, such as those mentioned above, increases in a prion-like manner.^271,280^ Thus, the physiological distribution of vital metabolites and nutrients is impaired.^269^ This adverse spread is the subject of current research and is considered to occur between regions that are synaptically connected.^271^ Highly active brain regions, such as the temporal lobes, are preferentially affected by the detrimental effects of arterial stiffening as well as early AD effects.^251^ Acute sleep deprivation can promote arterial stiffness after only one night and thus increase PWV.^281^ Unfortunately, sleep quality is also often impaired in cardiovascular disease.^271^ Obstructive sleep apnea syndrome (OSAS) has a special place in this regard, as there is a link to both reduced sleep quality and increased pulse waves and thus advanced arterial stiffness.^282^ This link is significant in terms of the importance of sleep to the glymphatic system and again leads to the suggestion of a mutually reinforcing mechanism.

In a study by Elias and colleagues,^283^ 409 subjects completed various physical and cognitive tests in which the correlation of cfPWV with age was related to various cardiovascular risk factors, mean arterial pressure (MAP), and demographic variables. The combination of increased PWV and advanced age resulted in poorer performance in global cognition, visuospatial organization and memory, verbal-episodic memory, scanning and tracking, and the abstract reasoning test. Working memory was not significantly associated with age or PWV, but this was probably due to failure to adjust the complexity of this test for the educational level of this population.^283^

### Kidney, gastrointestinal tract, testis, extremities and placenta

#### Kidney

In patients with end-stage renal disease (ESRD), the aortic PWV is a strong independent factor for all-cause and cardiovascular mortality.^284,285^ One study reported a significantly decreased likelihood of survival in hemodialyzed ESRD patients when PWV was not reduced by antihypertensive treatment.^27,286^

Compared with any other organ system, the kidney has the highest blood flow with the lowest precapillary resistance. Consequently, the tissue is vulnerable to pulsatile trauma and high blood pressure with glomerular damage, albuminuria, and decreased GFR.^27,67^ Aortic stiffness and thus a decrease in the impedance gradient, which results from the stiffness ratio of the aorta and muscular arteries, lead to a shift in pulse wave reflection further into the periphery, transferring pulsatile stress to the microcirculation.^33,234,287^ Under normal conditions, the small vessels and glomeruli are effectively protected by autoregulation, which consists of a myogenic response and tubulo-glomerular feedback. However, as soon as blood pressure persists outside the autoregulatory limits, remodeling of the resistance vessels occurs, leading to benign nephrosclerosis. When a certain pressure threshold is exceeded, vascular damage emerges with loss of autoregulation, and malignant nephrosclerosis manifests.^288^

EVA and arterial stiffening are observed mainly in CKD patients and in ESRD patients. Typically, outward remodeling of the arterial wall accompanied by hypertrophy and increased vessel radius occurs. At the forefront of the hardening process in these patients, a multifactorial genesis is considered, with calcification being an important component.^289,290^ In general, aortic stiffness and impaired renal function are related and were investigated in the CRIC study. An increase in cfPWV of 0.23 m/s for every 10 ml/min/1.73${m}^{2}$ decrease in eGFR was revealed, confirming that PWV is an independent predictor of progressive renal dysfunction. In addition, an elevation in the PWV of 2 m/s in each decade of life was found in diabetic patients compared with nondiabetic patients.^284,289,290^

New studies continue to be designed for further investigation of the link between CVD and CKD. Patients are more likely to die from CKD grade 4 than to experience disease progression to ESRD, mostly from cardiovascular disease.^287^ Cardiovascular risk in the presence of renal dysfunction cannot be fully explained by conventional risk factors^142^ and requires further research.

#### Gastrointestinal tract

The comorbidity of hypertension and metabolic dysfunction-associated steatotic liver disease (MASLD) is well known.^291–293^ The underlying pathophysiological mechanisms are not fully understood. Current research indicates that low-grade systemic inflammation induced by hepatocyte injury-associated secretion patterns is a potential mechanistic link.^294^

Furthermore, a bidirectional mechanism might accelerate liver fibrosis in MASLD as well as the progression of aortic stiffening.^295,296^ Villela-Noguiera and colleagues suggested that AGEs and subsequent NFκB activation and/or low adiponectin levels are potential causes of aortic stiffening and fibrosis.^296^

Interestingly, patients with inflammatory bowel disease (IBD) have an increased risk of cardiovascular diseases.^297–299^ In a comprehensive meta-analysis, Wu et al. revealed a concise association between endothelial dysfunction, elevated PWV, increased carotid IMT, and IBD. Once again, inflammatory patterns are considered key drivers.^298^ Accordingly, immunomodulatory therapy with anti-TNF agents leads to decreased cfPWV.^300^

#### Testis

Doppler studies have demonstrated that testicles also have low resistance to blood flow, making them susceptible to pulsatile stress. If damage occurs here due to a lack of regulatory capacity, structural degeneration occurs, and androgen production is restricted.^27^ In older men, increased PWV has been associated with low serum testosterone concentrations, raising the question of whether low androgen levels contribute to arterial stiffening.^301^ A study detected an elevation in PWV in patients with prostate cancer after treatment with anti-androgens, supporting the aforementioned hypothesis.^302^ In contrast, exogenous long-term high-dose administration of androgens in male-to-female transsexuals leads to significantly increased pulse wave velocities compared with those in transsexuals without hormone replacement.^303^ Conclusively, androgen levels that do not fit the frame seem to be potentially harmful because they promote further vessel stiffening.

#### Extremities

Growing evidence suggests that arterial stiffness is an independent risk factor for peripheral artery disease (PAD).^304,305^ In a large cohort study, 8960 participants were enrolled between 2008 and 2018. Starting with a baseline examination, these patients were followed until the incidence of PAD or the end of 2019. Overall, the highest incidence rate of PAD was linked to severe arterial stiffness, independent of hypertension or blood pressure. Current assessments for PAD do not include PWV measurements or surrogate arterial stiffness evaluation methods and neglect key predictive values.^304,306^

#### Placenta

The placenta has one of the largest blood flow resistance quotients and is therefore highly perfused. When the cardiac stroke volume increases to respond to elevated blood flow to sufficiently supply the fetus, arterial stiffness should also match a low level; otherwise, the PP will gain enormous magnitude and hence can cause vascular damage.^27^ In fact, the AIx, as an indirect indicator of arterial stiffness, decreases during the first and second trimesters and increases again in the 24th week and in the third trimester. Apparently, physiological pregnancies also undergo hemodynamic adaptations, with arterial compliance being influenced by the sex hormones progesterone and estrogen.^307^ The adaptation of maternal circulation appears to manifest in the first trimester and is held constant in the second trimester.^308^

Pathologic placental conditions such as preeclampsia or intrauterine growth restriction correlate with arterial stiffening and can be unmasked by predictors such as increased PWV, AIx or PP.^307,309,310^ In a longitudinal study by Robb et al.,^307^ preeclampsia patients presented a pathological increase in cfPWV and AIx during pregnancy. Interestingly, these parameters also persisted at elevated levels 7 weeks after delivery.

Another study shifted the focus to the AIx and attributed the lower correlation to the PWV and preeclampsia, suggesting that preeclampsia has a minor effect on arterial stiffness but leads to significant vasoconstriction and thus influences the AIx.^308^

### Retinal Microvascular Dysfunction

As mentioned above (mechanisms of vascular aging), endothelial dysfunction is an important determinant in aged, stiff arteries. One underlying key mechanism here is the absence of NO, which serves as one of the main vessel dilatators, especially in the microvasculature. The retinal vessel diameter is known to predict the risk for CAD and is related to cardiovascular and stroke mortality in middle-aged people.^311^ A novel method to noninvasively assess NO release in the endothelium has gained much attention in recent years. Dynamic retinal vessel analysis (DVA) uses flicker light to achieve more dilatation of the retinal vessels because of the greater amount of blood flow needed. There is valid evidence that impaired NO-mediated dilatation in response to flicker light is related to microvascular dysfunction and that DVA can serve as a marker for underlying CAD.^312^ Additionally, retinal arteriolar narrowing, venous widening and its ratio, the arteriolar-to-venous ratio (AVR), are linked to CAD, hypertension, and stroke mortality.^311,313^ A 4-year follow-up study by Lona et al. revealed an indirect relationship between AVR and PWV in prepubertal children, with the emphasis on arterial stiffness. Vigorous physical activity improved arterial stiffness after 4 years in this trial, most likely due to an increase in NO bioavailability and physiological laminar shear stress,^12^ leading to vasodilatation and extended blood flow.^313^

Barthelsmen and colleagues also reported a worsening of retinal microvascular dysfunction in patients with CAD and heart failure with reduced ejection fraction (HFrEF),^314^ whereas individuals with HFpEF have slightly better microvascular endothelial conditions. Hypertensive patients show only an intermediate level of impairment.^315^ Microvascular disease in the heart and retina is not strongly associated with conventional cardiovascular risk factors for macrovascular dysfunction, suggesting a distinct pathophysiology.^314,316^

Like in the brain, the autoregulation of retinal vessels also guarantees constant blood flow in a particular pressure range and protects the integrity of the vasculature and blood‒retinal barrier.^317,318^ Consequently, the similarity of microvascular pathologies in the brain and retina makes the examination of retinal blood vessels an attractive and easily accessible target for investigating brain vascular health.^319,320^ In the case of hypertensive retinopathy, persistent high blood pressure causes vascular remodeling, and IMT occurs.^321^ Thus, the lumen is narrowed, and consequently, the reduced blood flow leads to downstream ischemia. High pulsatility and aortic stiffness likely also play a role in retinopathy. If the pressure exceeds the compensatory tone, the muscle layer and the endothelium can be harmed.^321^ Furthermore, breakdown of the blood‒retinal barrier, ischemia, microinfarcts, and necrosis occur.^322^

In diabetic patients, arterial stiffness and PWV correlate with the progression of common complications, such as proliferative diabetic retinopathy, and hence emphasize the value of monitoring endothelial function in these individuals.^323^

### Summary: Arterial stiffness and resulting end-organ damage

1. **Systemic effects of arterial stiffness on several organs** Arterial stiffness leads to ISH and subsequently increased pulsatility, promoting a vicious cycle of microvascular damage in multiple organ systems (heart, brain, and kidney), primarily affecting organs with high blood flow and low resistance properties. Vascular remodeling and consequently disbalanced perfusion further downstream occurs. In general, vascular injury increases inflammatory mediator levels and neurohormonal dysregulation, promoting a vicious cycle of arterial stiffening and microvascular damage in all organ systems.
2. **Cardiac afterload and left ventricular remodeling** Arterial stiffening accelerates wave reflection and increases systolic pressure, myocardial wall stress, and myocardial oxygen demand, resulting in left ventricular hypertrophy (LVH) and diastolic dysfunction (e.g., heart failure with preserved ejection fraction, HFpEF).
3. **Cerebral microcirculation and cognitive decline** Excessive pulsatile stress leads to increased shear forces on the microvasculature of the brain, contributing to the formation of white matter lesions, microinfarcts, and blood–brain barrier disruption. These pathophysiological events predispose patients to vascular cognitive impairment and dementia.
4. **Kidney dysfunction and CKD progression** Renal circulation is particularly susceptible to heightened systolic pressures arising from a loss of aortic compliance. Prolonged exposure to high systolic pressure induces glomerular damage, albuminuria, and progressive chronic kidney disease.

## Clinical measurement of arterial stiffness

In 2007, the European hypertension guidelines first recommended the measurement of arterial stiffness.^324^ The 2023 ESH hypertension guidelines and the 2024 ESC hypertension guidelines recommend PWV measurement for stratifying the risk of patients with hypertension.^325,326^ Compared with conventional risk-based scores, especially in young and middle-aged patients with a low or moderate risk for cardiovascular events, PWV measurements are recommended to more precisely evaluate individual risk, as PWV measurements provide greater benefits.^326^

The 2017 ACC/AHA hypertension guidelines recommend the use of the PWV alongside the carotid intima–media thickness and coronary artery calcium score as potential noninvasive methods for detecting organ injury and atherosclerosis.^327^

Central systolic blood pressure (cSBP), Aix, and PWV are better predictors of cardiovascular risk and mortality than peripheral blood pressure is.^31,67,328^

However, the clinical use of arterial stiffness measurement is limited because of heterogeneous methods and a lack of reference values.^329^

There are several invasive and noninvasive methods for measuring arterial stiffness. Here, we briefly present the principles and pros/cons of the different methods (Table 1).

**Table 1:** Overview of different methods and devices used to measure the PWV.^154^

Device	Value	Method	Invasiveness	Procedure	Pros	Cons
**Pressure catheter**	Aorta PWV	Direct	Invasive	• Pigtail catheter is placed aortal, • Pressure is recorded at aorta ascendens and at the bifurcation • Distance is measured and time interval is determined	• Well-specified phenotype when both catheters are positioned within the aorta • Gold standard method for PWV • High temporal resolution • Includes aorta ascendens	• Invasive • Expensive • Confined to cath-lab • Only in patients scheduled for catheterization/validation • No direct view on aortic anatomy and position of catheters
**MRI**	Aorta PWV	Direct	Noninvasive	In one examination: • 3D imaging of aorta • Path length (anatomic imaging) • Transit time (phase-contrast sequences)	• Accurate measurement in accordance to anatomic conditions • Includes aorta ascendens • Noninvasive • Well-specified phenotype	• Expensive • Radiology department required • Not appropriate for patients with claustrophobia, metallic implants, children or mentally handicapped people • Temporal solution is limited
**Sphygmocor, Complior**	Carotid-femoral PWV	Direct	Noninvasive	• Recording pulse wave at carotis and femoralis via pressure transducer (sphygmocor) or piezo transducer (complior) • Synchronizing R-wave with the two pulse waves • Determining transit time	• Relatively inexpensive • Best proxy for aorta PWV • Well validated, cut off values for end organ damage • Noninvasive gold standard • Applicable for everyday use	• Inaccurate external pathway measurement • Trained staff required • Aorta ascendens and aortic arch excluded
**VaSera VS-1000, BoSo ABI100**	Brachial-ankle PWV	Direct	Noninvasive	• Collecting pulse wave signal via blood pressure cuffs at brachial and ankle arteries	• Applicable for everyday use • Inexpensive, fast, automated • Accuracy less dependent from user • CAVI: arteriosclerosis scale and cut off values available/blood pressure independent • Widely used and validated in Japan	• Includes unwanted muscular arteries • Less accordance to aortic PWV • Ambiguous phenotype
**Arteriograph**	Aorta PWV	indirect	Noninvasive	• Oscillometric, single cuff • Recording of systolic and reflected systolic pulse wave • Measuring jugulum-symphysis distance and time from first to second wave peak • Applicate formula	• Easy applicability and high potential for everyday use • Fast and inexpensive • Strong correlation to invasive obtained values • Automated, user-independent	• Arguable physical model (single tube with single point of reflection in bifurcation) • Aorta ascendens excluded

### Invasive measurement

The crucial structural changes in the arterial wall causing the aforementioned negative effects affect primarily the aorta and can be quantified by the aortic PWV. In an invasive procedure, a pigtail catheter is placed aortally, and the position is checked by fluoroscopy. The system is now capable of recording pulse pressure curves in the ascending aorta and at the level of its bifurcation. The points are marked with tape. After removal of the catheter, the distance traveled by the pulse wave is measured, and a time interval is determined.

### Noninvasive measurement

The noninvasively measured cfPWV is an adequate proxy of invasively measured aortal PWV (aoPWV) and is easier to perform with regard to complications and comorbidities.^330,331^ To calculate the cfPWV correctly, the distance between two points of the arterial tree, in this case, the carotid and femoral arteries, is measured and divided by time, and the pulse wave needs to pass this distance. The prevailing system pressure at the time of measurement must always be considered. The average velocities in the ascending aorta are approximately 4–5 m/s, 5–6 m/s in the abdominal aorta and 8–9 m/s in the iliac and femoral arteries. The diverse values result from deviating compliance of the arterial wall in different parts of the aorta; hence, a stiffness gradient can be determined.^332^ The current expert consensus recommends a threshold of 10 m/s for the diagnosis of arterial stiffness.

A well-validated and frequently used method is tonometric measurement of the pulse wave via pressure transducers on the carotid and femoral arteries. Additionally, an ECG is required to synchronize the R wave with the two pulse waves. Afterwards, the transit time is determined via the intersecting tangent method (ITM). Other tonometric approaches follow the measurement of both arteries simultaneously or with a piezoelectric transducer.^28,156^ The latter is clinically very well validated and one of the most reliable devices for measuring PWV.^156,331^ The simultaneous version uses the foot-to-foot method, which illustrates the beginning of the pressure curve rise at the end of diastole as a foot.^67,331^

Overall, uncertainty emerges regarding the external measurement of the length of the aorta compared with the intravascular measurement.^156^ Since the true aortal length remains unknown without invasive measurement, noninvasive methods can only approximate this value. It appeared that multiplying the difference in the distance between the carotid and femoral measurement points by a factor of 0.8 seems close to the value invasively obtained. Owing to this adjustment, the threshold of 12 m/s was reduced to 10 m/s.^332,333^ Nevertheless, there are a few disadvantages related to the cfPWV: on the one hand, the precise recording of the femoral pressure curve is problematic in patients with metabolic syndrome, obesity, and peripheral artery disease (PAD); on the other hand, stenosis of the aorta, iliac artery, or proximal femoral artery may limit wave progression. In addition, obesity or chest size can lead to measurement errors in distance.^67^

An alternative way is to collect the pulse wave signal via blood pressure cuffs on the brachial artery and ankle arteries. The so-called brachial-ankle PWV (ba-PWV) has its own diagnostic qualities but includes the muscular leg arteries, which are quite irrelevant for the consequences of arterial stiffening.^154,238,332^ Therefore, an adjustment of the threshold value for this measurement method to 16 m/s is needed.^332^ The benefits are less dependence on the user and his or her accuracy, easy handling of patients with obesity, better reproducibility and applicability, and the possibility of long-term use.^238^ A conversion formula, obtained from a study with just 44 patients, highlights the correlation of both measurement methods.^156,334^ Prognostic data for the cfPWV are more comprehensive, yet baPWV is highly regarded and widely used in Japan, for example. A 2008 study revealed better correlations between baPWV and left ventricular mass, diastolic function, and arterial stiffness parameters such as effective arterial elasticity than between baPWV and cfPWV.^156^ Both values are nevertheless dependent on blood pressure. However, baPWV is a general vascular biomarker and not a specific marker for arterial stiffness. To circumvent this problem, the CAVI was created as a stiffness and arteriosclerosis indicator for the thoracic and abdominal aorta and the iliac, femoral and tibial arteries. This parameter appears to be suitable for screening individuals with moderate to severe arteriosclerosis. A dimensionless value below 8.0 is considered normal, a value between 8 and 9 is borderline, and a value above 9 is suspicious for arteriosclerosis.^335^ However, all of the previously mentioned noninvasive PWV assessment methods neglect the ascending part of the aorta and, proportionally, the subsequent aortic arch, which are arguably the most distensible elements of the arterial tree.^27,154,287^

Indirect noninvasive measurements of the PWV are based on aorta-bifurcation-reflection. The systems allow user-independent measurements to be easily applied and have high potential for everyday use. The system uses a single upper arm cuff and pumps it up to 35 mmHg above the current systolic blood pressure so that the local arterial blood flow fully stops. Under these conditions, a sensitive pressure sensor in the cuff can detect pulse pressure differences across compressed tissue. The first peak of the pulse curve is caused by the direct systolic wave, the second is caused by the reflected systolic wave, and the third is caused by the diastolic wave. The device records the reflection of the pulse wave, which, according to Horvarth et al.^,^^336^ occurs predominantly at the aortic bifurcation. Afterwards, the distance between the jugulum (Jug) and symphysis (Sy) as well as the time difference between the first two peaks (RT) is measured. Thus, the aortic PWV can be determined via the following formula:

$${aoPWV}\left(\frac{m}{s}\right)=\frac{{Jug}-{Sy}(m)}{{RT}/2(s)}$$

In addition to the PWV, several systems can measure and calculate brachial and central blood pressure as well as the AIx within two to three minutes. All the values are significantly correlated with the invasively measured values, as shown in the publication by Horvarth and colleagues.^336^ In this study, an average aortic PWV of 9.41 ± 1.8 m/s was found invasively in 22 subjects, whereas noninvasive measurement was able to determine a mean value of 9.46 ± 1.8 m/s. Thereafter, the validation was assessed as Grade B using the British Hypertension Society classification, and its clinical applicability was described as acceptable. A weakness of the principle applied here is the assumption that the arterial system is based on a single-tube system (aorta) with a single point of reflection (bifurcation).^154,337^

Furthermore, focusing on a much more effortful method, MRI provides an extremely accurate measurement of PWV. The correct path of the pulse wave can be determined in accordance with anatomical conditions. The aortic arch and ascending aorta, sections with pronounced distensibility, can also be assessed via this method, but good temporal resolution is required because of the short transit time of the pulse wave.^154^ The cumbersome nature of the examination does not yet permit its proper use.^154,331^

Ultrasound devices allow noninvasive evaluation of the elastic properties of the arterial wall. The B- and M-modes can be used for local stiffness assessment and PWV evaluation by measuring diameter changes as well as pressure variations in the ascending or descending aorta and visualizing wall strain. Doppler measures blood flow and, in various arteries, such as carotid and femoral arteries, regional PWV.^331^

However, one central limiting factor for the implementation of PWV in clinical practice is the use of several methods for assessing PWV and varying reference values. In this context, a current systematic review meta-analysis of 167 studies with 509,743 participants revealed global and regional age- and sex-dependent distributions and reference values of baPWV and cfPWV for healthy adults.^338^ In this regard, it is important to note that PWV values are dependent on the method used. Nevertheless, these data could increase the clinical use of PWV.

## Prevention and therapy of vascular aging and arterial stiffness

Cardiovascular disease often originates in childhood, and the cumulative burden of risk factors manifests later in life in diseases for which only damage control or symptomatic treatment is usually possible. This finding highlights the importance of early biomarkers (such as arterial stiffness) to identify individuals at risk for (early) vascular aging and to tailor, personalized preventive and therapeutic approaches. In particular, vascular screening in childhood/young adults could help individuals with EVA.

Central key elements of prevention and therapy for vascular aging are restrictive control of cardiovascular risk factors (e.g., blood pressure management, lipid management, and no smoking), adherence to a healthy diet, and physical activity. This approach needs an intensive combination of personalized medicine in combination with a strategic public health initiative (e.g., smoking bans and the promotion of healthy lifestyle factors throughout the lifespan).

### Prevention

Vascular aging and arterial stiffness are the results of lifelong processes that probably begin in utero.^339^ They progress and are aggravated over decades by exposure to multiple cardiovascular risk factors (e.g., genetics, physical inactivity, overweight, smoking).^340^ Recent results from preclinical and clinical studies highlight the potential of lifestyle modifications (e.g., physical activity, calorie restriction, intermittent fasting) for cardiovascular prevention and slowing the arterial stiffness continuum.^341^ It is hypothesized that the pharmacological modulation of vascular aging, particularly exercise mimetics, has the potential to prevent end-organ damage associated with the arterial stiffening process.^86,342–344^

Preventive approaches seem most effective in patients at risk for vascular stiffness.

EVA in particular can be attributed to dietary and exercise habits in childhood and is correlated with a variety of cardiovascular risk factors.^345^ Lifestyle interventions should be considered and implemented in exercise and dietary plans.^345^

#### Physical activity and exercise

Physical activity and/or physical exercise is a low-cost intervention for primary and secondary prevention of several noncommunicable diseases (e.g., cardiovascular diseases, dementia, and diabetes).^346^ In this context, “physical activity” is defined as any muscle-induced bodily movement that increases energy expenditure above ~ 1.0/1.5 metabolic equivalent of a task (MET, 1 MET = 1 kcal (4,184 kJ) × kg−1 × h − 1), whereby “physical exercise” is a specific, planned and structured form of physical activity.^347^ The World Health Organization (WHO) recommends at least 150 minutes of moderate-intensity or 75 minutes of vigorous-intensity aerobic activity and additional strength training per week.

A systematic review and meta-analysis revealed that aerobic exercise can improve arterial stiffness.^348^ Moreover, they reported that higher exercise intensities and initial greater arterial stiffness are associated with more pronounced effects. Positive effects of physical exercise on arterial stiffness were also reported in a systematic review and meta-analysis of patients with hypertension.^68^ Additionally, Lan et al. reported different effects of exercise on arterial stiffness depending on age.^349^ This could indicate the necessity of specific tailored exercise prescriptions for vascular prevention across the lifespan.

Shibata et al. reported dose-dependent effects of lifelong exercise training on arterial stiffness,^350^ indicating that master athletes who trained near-daily over long time periods have more compliant central arteries than their sedentary peers do. In contrast, some authors reported increased arterial stiffness in athletes with high exercise doses. For example, Vlachopoulos et al. reported greater arterial stiffness in marathon runners than in active, healthy controls.^351^ Potential mechanisms of increased arterial stiffness in high-trained athletes could be repetitive high blood pressure levels (exercise hypertension), leading to fibrosis^352,353^ and/or increased sympathetic activation.

However, especially in the context of the current results from the Master@Heart study^,^^354^ future studies are needed to investigate the potential dose-dependent effect of physical exercise on vascular aging and arterial stiffness. To date, the Master@Heart is the largest and most comprehensive study investigating the dose‒response relationship between exercise and coronary atherosclerosis. The results of this study indicate that lifelong endurance athletes have more coronary plaques but fewer CVD events because of plaque stability.

The underlying mechanism by which physical activity affects arterial stiffness is still not fully understood. In general, physical activity has pleiotropic effects on vascular health.^355–359^ It reduces chronic low-grade systemic inflammation, cellular senescence, ET-1 signaling, TGF-β activity, and endothelial dysfunction and has a positive influence on several risk/modulating factors (e.g., hypertension and obesity).^14,360–365^

#### Diet

Animal and human studies indicate that caloric restriction without malnutrition is one of the most powerful interventions for increasing lifespan and preventing several age-related chronic diseases.^366,367^ Caloric restriction is associated with reduced low-grade systemic inflammation, reduced adipokine-induced immune activation, reduced oxidative stress, reduced sympathetic and renin‒angiotensin system activation and several other pleiotropic effects.^366,367^

Intermittent fasting and ketogenic diets are further potential strategies to mimic the effects of caloric restriction.^368^ Randomized controlled trials have shown beneficial effects of both dietary interventions on cardiovascular surrogate parameters.^369,370^ However, the effects of dietary interventions on arterial stiffness are still largely unknown.^371^ Studies with small sample sizes suggest potential beneficial effects of different macronutrient compositions (e.g., polyunsaturated fatty acids and polyphenols). The consumption of Ω-3 fatty acids (fish oil)^73^ and soy isoflavones,^372^ as well as consistent salt restriction, also has a positive effect on vascular wall distensibility.^11,373,374^ Alcohol displays a bipolar effect, according to Paracelsus: “It is the dose alone that makes a thing not a poison”. With low to moderate alcohol consumption, a decrease, and with excessive alcohol consumption, an increase in the PWV could be detected. The beneficial effect is probably due to the influence on HDL cholesterol.^11,375,376^ An interesting study by Vlachopoulos et al. revealed advantages in terms of vascular stiffness, the wave reflection index, and aortic pulse pressure with increased cocoa consumption (>4.63 g/d).^377^ In 2012, Johansen and colleagues linked predictors such as waist circumference, heart rate, and interleukin-1 status to pulse waves in men, whereas in women, there were more correlations with adiponectin, triglycerides, and pulse pressure. For both sexes, the waist‒to-hip ratio, obesity and elevated serum inflammation could be identified as predictors of PWV progression.^378,379^

### Therapy

The basic component in the management of arterial stiffness is controlling cardiovascular risk factors. Currently, the most powerful intervention is related to a reduction in blood pressure.^332,373^ Stiffness is improved by reducing transmural wall tension.^332,380^ Several randomized, controlled trials have shown that angiotensin-converting enzyme (ACE) inhibitors, calcium antagonists, ß-blockers, and diuretics can significantly reduce arterial stiffness.^381^ According to current guidelines, initial antihypertensive drug therapy should consider a dual combination of an ACE inhibitor or angiotensin receptor blocker with a calcium channel blocker or diuretic (optimal single pill). Moreover, personalized treatment should be based on individual factors and comorbidities (e.g., atrial fibrillation).

As mentioned, ACE inhibitors are the first-line option with experimentally proven effects on fibrosis, collagen, and the ECM.^332^ Additionally, the anti-inflammatory and anti-VSMC proliferative properties of RAAS blockers also contribute to decreased arterial stiffness; more precisely, ACE inhibitors and angiotensin receptor blockers (ARBs) increase angiotensin 1-7 levels while lowering Ang II levels, thus causing the aforementioned effects.^132,382,383^

Current results from the MAGMA trial (MR Antagonism Clinical Evaluation in Atherosclerosis) show that spironolactone can prevent the progression of arterial stiffness.^384^

A promising new therapeutic target in the RAAS appears to be the PRR (prorenin receptor), which is associated with VSMC proliferation, neovascularization, endothelial dysfunction, and cardiovascular disease progression.^86,385^

Studies in humans and animals have demonstrated reverse remodeling in small and large arteries.^386^ The biochemical background probably comprises a modification of the α5β1-integrin-fibronectin complex, which plays a crucial role in regulating arterial elasticity via transmembrane signaling.^386,387^ Dissociation of the aforementioned complex caused by RAAS inhibition is associated with a significant and selective decrease in arterial stiffness and PP. The application of tandolapril, e.g., leads to fibronectin accumulation without binding to its receptor integrin, hence lowering cell wall stiffness.^388^

Remodeling to more elastic forms of the vessel wall occurs as the result of prolonged pressure relief following consistent hypertension treatment, especially in terms of RAAS inhibition.^332,386^

The application of conventional β-blockers should be considered cautiously if there is not a given essential indication, such as atrial fibrillation, or if tachyarrhythmia after myocardial infarction is prevented because of potential negative effects on PWV and central blood pressure levels. Recently, some meta-analyses by Koumaras and colleagues revealed potentially less favorable effects of atenolol on central pulsatile hemodynamics. Despite the observed decreases in peripheral blood pressure and PWV, the AIx and central pulse pressure (PP) did not change significantly. This issue is most likely caused by the heart rate-lowering effect of atenolol, leading to the return of the reflected pulse wave earlier in the cardiac cycle and hence during systole.^389^

On the other hand, several studies with vasodilating β-blockers such as nebivolol or carvedilol suggest favorable effects in terms of arterial elasticity, likely due to an increase in the bioavailability of NO.^389,390^ Different pharmacological studies have revealed increasing evidence that long-term treatment with ACE inhibitors, calcium channel blockers, and some β blockers is generally similar, so the effectiveness of antihypertensive agents in the treatment of arterial stiffness in some cases may be related to the individual genetic background.^391^ A potential negative effect of vasodilating agents is the increase in forward pressure wave amplitude connected to stronger penetration of excessive pressure and flow pulsatility into the microcirculation.^162^

Several studies have investigated the effects of HMG-CoA reductase inhibitors (statins), with the sobering result that such drugs tend to affect the arterial stiffness of muscular arteries rather than that of the aorta and are therefore used less in this context.^11,392^ In addition to lowering LDL-C, the anti-inflammatory effects of statins are useful in inflammatory processes that contribute to aortic stiffness. Positive effects are observed here with atorvastatin in the setting of CKD or rheumatic arthrosis (RA).^392^ The quotient of triglycerides and HDL-C represents an independent risk factor for cardiometabolic diseases and shows direct proportionality with PWV, which could not be proven for the respective individual values.^393^ Nevertheless, statins have been shown to have beneficial effects on arterial elasticity in different meta-analyses. Interestingly, their pleiotropic impact seems to be independent of blood pressure alterations, lipid profiles, and even statin type. Endothelial dysfunction is improved by inhibition of the Rho/ROCK (Rho-Kinase) pathway, leading to increased NO production, while antithrombotic properties such as atherosclerotic plaque stabilization are also attributed to this drug class.^394^ Evolocumab, an injectable lipid-lowering drug, apparently decreases arterial stiffness by inhibiting proprotein convertase subtilisin/kexin type 9 (PCSK9), thus minimizing oxidative stress and inflammation.^395^

### Emerging medical therapies

Several approaches are emerging to improve antistiffness drug therapy by targeting underlying pathophysiological mechanisms (Table 2). Roth et al. provided a comprehensive review of the pharmacological modulation of vascular aging and arterial stiffness.^86^ The limitations of novel drugs include preclinical methodological considerations and interactions with lifestyle factors (e.g., physical exercise, smoking, diet) and/or sex.

**Table 2:** Overview of the arterial stiffness mechanism, resulting consequences, and potential therapeutic interventions

Mechanism	Consequence of dysregulation	Potential intervention	Reference
VSMC phenotype switch	• Variety of VSMC phenotypes with different characteristics • Providing inflammatory environment • Migration • Calcification • Apoptosis • Proliferation • ECM remodeling • ROS production • Contractile type • Vascular fibrosis	• ACE inhibition • Mi-RNA92a inhibition • Echinatin	^14^^,423^^,424^
Low-grade systemic inflammation	• Direct reduction of eNOS caused by inflammatory mediators (e.g., TNF-α, IL-6, and CRP) • Endothelial dysfunction • DNA damage • Involved in various vicious cycles concerning ROS, RAAS	• Treatment of hypertension and obesity • Physical activity • PCSK9 inhibition • Statins • ACE inhibition/ARB • Interleukin-6 inhibition • Inhibitors of the NLRP3 inflammasome	^14^^,133,139,236^^,^^361^^,364^^,365,378,395,425^
Oxidative stress	• Lipid oxidation • Endothelial dysfunction • DNA oxidation • Inflammation • Impaired plaque stability • Cellular senescence • Enhancng ROS production by uncoupling eNOS	• SGLT-2 inhibition • GLP-1 R agonism/GLP analogons • Antioxidants • Statins • ACE inhibtion • PCSK9 inhibition	^86^^,395^^,^^426–430^
NO-cGMP-PKG	• Vasoconstriction • NO inhibits migration and proliferation of VSMC, neointima formation and ECM alterations • cGMP signaling has been implicated in vascular remodeling as an anti-fibrotic and anti-hypertrophic messenger system	• NO donors • SGLT-2 inhibition • BH4 supplementation • SIRT1 activators • Phosphodiesterase inhibition	^426^^,429^^,431–435^
ECM remodeling	• Dysbalanced elastin/collagen ratio—altered mechanistic properties • EDP mediated inflammation and calcification	• ${{\alpha}}-{\rm{ketonamides}}$ • Minoxidil • NEU-1 inhibition	^158^^,397–402^
Calcification	• VSMCs as the main actor provide calcifying mileu in tunica media • Pivotal contribution to decreased distensibility • Increased risk of infarction and cardiovascular mortality	• TNAP inhibtion • NR4A3 inhibtion	^404^^,405^
Cellular aging	+Perivascular inflammatory signaling • Structural and functional wall changes • Decreased NO-bioavaibility • Apoptosis • Endothelial dysfunction • Vascular remodeling	• Senolytics • Senomorphics	^172^^,403^
RAAS	• Vascular fibrosis and inflammation • Vascular remodeling • ROS production	• Bariatric surgery • PRR inhibition • ACE inhibition/ARB	^86^^,332^^,382,383,385,436^
PVAT signaling	• Loss of vasodilatory, anti-inflammatory, and antioxidant effects on the vasculature • Perivascular inflammatory state provided by adipocytes and infiltrative immune cells • Altered differentiation capacities and loss of PGC1α in aged PVAT-derived stromal cells leading to vascular remodeling	• PPAR$\gamma$ activation • Pioglitazone, • Caloric restriction/diet	^143^^,144,148,437,438,439^

Potential therapeutic targets are low-grade systemic inflammation, oxidative stress, NO signaling, RAAS signaling, ECM remodeling, calcification, VSMCs, and cellular senescence.^27^ Several drugs have been validated in clinical trials but not in the area of arterial stiffness.^33^

Hypomethylation of microRNA-203, for example, downregulates the expression of the FA adhesome, leading to ECM remodeling and increased VSMC stiffness via an altered FAK/Src pathway^33^. Thus, epigenetics and ECM remodeling are identified as potential targets for intervention. Epigenetic reprogramming is considered one of the major keys to preventing EVA. Epidrugs can regulate the chromatin condensation state via DNA methylation or by targeting noncoding RNAs.^33,396^

Another important approach is to prevent elastin degradation and/or ECM remodeling. Cathepsin S, a potent mammalian elastase leading to elastin breakdown, can potentially be suppressed by α-ketonamides.^397^ Long-term administration of minoxidil preserves integrity and induces neosynthesis of elastin, hence securing the biomechanical properties of the aorta.^398^ In particular, bioactive EDPs and their adverse effects via ERC signaling are promising new targets. Currently, in vivo studies are investigating the application of NEU-1 inhibitors or other potential drugs.^158,399–401^ For example, C9-BA-DANA and CG17701 significantly delayed fatty streak formation in the aortic root of mice.^402^

A pivotal role in mitigating EVA involves the use of so-called senotherapeutics. These drugs can be classified into senolytics, which are capable of selectively detecting and eliminating senescent cells, and senomorphics, which are dedicated to modulating cellular functions or delaying aging progression.^403^

Given that vascular calcification is a cornerstone in preventing arterial stiffness, promising data suggest that the inhibition of tissue nonspecific alkaline phosphatase (TNAP) decreases cardiovascular calcification.^404^ Additionally, the latest research identified nuclear receptor subfamily 4 group A member 3 (NR4A3) as a mediator of histone lactylation and epigenetic signaling that participates in the pathogenesis of medial arterial calcification. The downregulation of NR4A3 prevents osteoblast differentiation-related gene expression, reduces vascular calcium storage, and preserves the contractile subtype of VSMCs, making NR4A3 a novel target for the treatment of CVD.^405^

## Conclusion and future perspectives of arterial stiffness research

Cardiovascular diseases are the leading cause of morbidity and mortality worldwide and substantially contribute to the global disease burden. One significant underlying mechanism is arterial stiffness, with resulting structural and functional vascular alterations. On the basis of impaired cushioning function, especially of large arteries, arterial stiffness causes (i) ISH, (ii) reduced coronary perfusion and increased afterload with resulting left ventricular diastolic dysfunction, and (iii) microvascular alterations with resulting end-organ damage (e.g., HFpEF, vascular dementia, and chronic kidney disease).

Central risk factors for arterial stiffening are aging, hypertension, truncal obesity, and a sedentary lifestyle. Current research indicates that arterial stiffness is one of the earliest markers of vascular aging and is a predictor of CVD independent of traditional risk factors such as smoking, hypertension, hypercholesterolemia, and diabetes. In addition, arterial stiffness, measured as PWV, is a potential early biomarker for arteriosclerosis and EVA and, as such, could represent a high-priority target for cardiovascular prevention. Although current guidelines (ESH and ACC/AHA) recommend the measurement of arterial stiffness for personalized risk stratification of patients with hypertension, a better understanding of its pathophysiology is needed to define new targets for prevention and to bring us closer to healthy vascular aging.

**Fig. 8:**
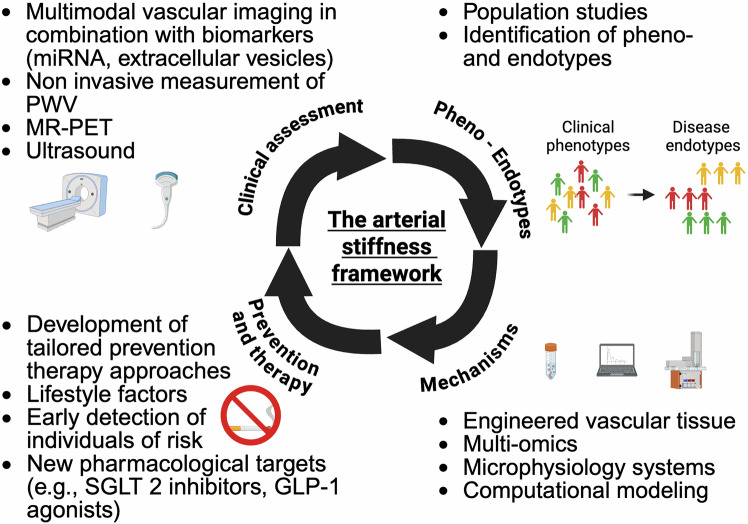
Arterial stiffness framework. Future translational arterial stiffness research should (i) enhance clinical assessment, (ii) identify specific phenotypes and endotypes, (iii) decipher the underlying mechanisms and “new” risk factors (e.g., chronic psychosocial stress, air pollution), and (iv) develop tailored prevention and therapy approaches. Created with BioRender.com

We suggest that translational arterial stiffness research should (i) improve clinical assessment, (ii) identify specific pheno- and endotypes, (iii) decipher underlying mechanisms and “new” risk factors (e.g., chronic psychosocial stress, air pollution), and (iv) develop tailored prevention and therapy approaches (Fig. 8).

### Clinical assessment

To date, the clinical use of arterial stiffness measurement is limited because of heterogeneous methods (e.g., baPWV, cfPWV) with varying and/or missing reference values. In this context, we propose the measurement of large artery stiffness (e.g., cfPWV). The measurement of regional large artery stiffness should be expanded in the future for pathomechanistic studies via a multimodal vascular imaging approach in combination with vascular biomarkers (e.g., extracellular vesicles, blood biomarkers for inflammation, and endothelial dysfunction). Multimodal vascular imaging (e.g., combined MRI and PET, ultrasound, and CT) can combine the strengths of different imaging modalities and provide new insights into vascular aging mechanisms. In dementia research, multimodal brain imaging (especially MR-PET) has been used for years to follow the spatiotemporal development of neurodegenerative diseases.^406^ Specific PET tracers for amyloid and tau in combination with high-resolution MRI play a central role in dementia research and the development of specific pharmacological approaches.^407^

For vascular aging, specific PET tracers for fibrotic remodeling (e.g., FAPI-PET), inflammation (macrophage-PET), and sympathetic tone (norepinephrine-PET) could provide new insight into the mechanisms and spatiotemporal development of arterial stiffness.^408^ Furthermore, CT imaging could reveal specific calcification patterns associated with arterial stiffness.^409,410^

In addition, owing to technological advances, wearables (e.g., smartwatches and Oura ring) can be used for blood pressure monitoring and pulse wave analysis. These findings could enable the study of arterial stiffness in large clinical trials and population studies. In addition, wearables combined with computational modeling and machine learning approaches provide new insights into the understanding of arterial stiffness (e.g., age effects, sex effects, and modulating factors).^411–414^ In this context, current research indicates that a finger ring can estimate aortic arterial stiffness via machine learning approaches.^415^

### Identification of phenotypes and endotypes

Arterial stiffness is a clinical syndrome caused and amplified by several modifying and/or modulating factors (e.g., obesity, CKD, diabetes, and smoking).^23,204,209,218,222,231,282,287^ Phenotypes are characterized by observable characteristics (e.g., obesity-related arterial stiffness phenotype), whereas endotypes are characterized by distinct pathophysiological mechanisms.^416,417^ In particular, the endotype approach could offer new perspectives for the diagnosis, monitoring, and treatment of arterial stiffness. Thus, population studies with specific clinical assessments and the use of artificial intelligence are needed for the identification of phenotypes and endotypes. Using multimodal imaging (e.g., CT imaging), specific endotypes (e.g., media calcification endotypes classified by the Agatson score) can be classified.^418^ Additionally, population studies could identify the role of “new” cardiovascular risk factors on arterial stiffness (e.g., air pollution, chronic psychosocial stress). In this context, wearable-based arterial stiffness measurements provide a cost-effective approach for cardiovascular monitoring.

### Deciphering the underlying mechanisms and “new” risk factors for arterial stiffness

Novel methods in experimental and human research will help to identify the molecular and cellular mechanisms of arterial stiffness in depth. The use of engineered vascular tissue, multiomic approaches, microphysiological systems, and computational modeling will provide insights into the underlying pathophysiology.^33^ Advances in specific microfluid cell culture systems (e.g., microfabricated fluid channels and microfabricated blood vessel systems) will provide new insights into the cellular mechanisms of arterial stiffness.^171^ In particular, multiomic data are promising tools for the identification of tailored biomarker-based interventions.^419^ In this context, AI-powered applications include multiomic data analysis, biological network construction, identification of potential diagnostic, predictive and prognostic biomarkers, clinical trial success prediction, development of personalized prevention and therapy interventions, safety monitoring and automated adverse event monitoring in clinical trials.^420^

### Development of tailored prevention and therapy approaches

In particular, the identification of pheno- and endotypes and underlying mechanisms in combination with AI-powered applications will help in the development of tailored preventive and therapeutic approaches for arterial stiffness. Additionally, future studies should investigate the effects of lifestyle factors and “novel” pharmacological approaches (e.g., SGLT-2 inhibitors, GLP-1 analogs) on arterial stiffness. In this context, experimental data suggest that SGLT-2 inhibitors may reduce arterial stiffness, although the mechanism is still not known.^421–425^

### Future directions

Future translational arterial stiffness research should (i) enhance clinical assessment (e.g., multimodal imaging, wearable-based PWV analysis in large clinical trials and population studies), (ii) identify specific phenotypes and endotypes, (iii) decipher the underlying mechanisms of the use of state-of-the-art technologies (e.g., specific microfluid cell culture systems, machine learning approaches) and “new” risk factors (e.g., chronic psychosocial stress, air pollution), and (iv) develop tailored prevention and therapeutic approaches (e.g., new pharmacological agents targeting low-grade systemic inflammation, AI-based personalized intervention). In this context, we call for an interdisciplinary and translational framework for further research into arterial stiffness.
